# *Staphylococcus aureus* biofilm-associated component PNAG stimulates the secretion of the immunomodulatory chemokine CXCL10 via Dectin-1 signaling

**DOI:** 10.1038/s42003-025-08503-z

**Published:** 2025-07-31

**Authors:** Reza Gheitasi, Daniel Weiss, Mario M. Müller, Karolin Sommer, Daniela Röll, Alexander Mosig, Mathias. W. Pletz, Oliwia Makarewicz

**Affiliations:** 1 Institute of Infectious Diseases and Infection Control, Jena University Hospital, Friedrich Schiller University, Jena, Germany; 2 Septomics Research Center, Jena University Hospital, Jena, Germany; 3 Functional Proteomics, Jena University Hospital, Jena, Germany; 4 Institute for Biochemistry II, Jena University Hospital, Friedrich Schiller University, Jena, Germany; 5 Integrated Research and Treatment Center - Center for Sepsis Control and Care (CSCC), Jena University Hospital, Jena, Germany

**Keywords:** Bacterial infection, Biofilms

## Abstract

*Staphylococcus aureus* is a common human pathogen associated with many infections. The key factor contributing to the virulence of *S. aureus* is its ability to form difficult-to-treat and recalcitrant biofilms. One of the major staphylococcal biofilms matrix compounds is poly-*N*-acetylglucosamine (PNAG). In previous study, we observed an increased secretion of various cytokines and chemokines when immune cells were stressed by *S. aureus* biofilms. In this study, we aimed to analyze the effect of PNAG on the secretion of the CXCL10 chemokine subfamily by peripheral blood mononuclear cells and monocytes and studied the connection to the Dectin-1-Syk-CARD9 signaling pathway, as Dectin-1 is the major pattern recognized by polysaccharide structures. We showed that, in contrast to the major virulence factor surface protein A, PNAG primarily elevates the secretion of CXCL10. This secretion was interrupted by blocking the Dectin-1 receptor or tyrosine kinase Syk. PNAG exposure resulted in increased Dectin-1 and CARD9 expression as well as increased NF-κB and CXCL10 expression, which may be related to the long-term memory processes of T cells. We also showed that PNAG induces the formation of CD14 + CXCL10+ monocytes that can migrate to the site of infection, triggering an innate immune response against *S. aureus*. This study provides insights into the complex interaction of the staphylococcal biofilms matrix with immune chemotaxis and shows that immunologic processes leading to bacterial infections should be viewed in a more differentiated manner, as biofilms are the preferred formation of microorganisms.

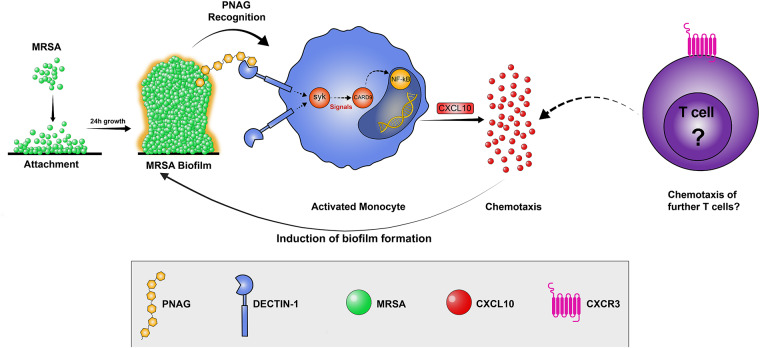

## Abstract (web summary)

*S. aureus* biofilms stimulate CXCL10 chemokine secretion, via the biofilm matrix compound PNAG, which also stimulates NF-κB, Dectin-1 and CARD9 expression, inducing monocyte formation, in turn triggering immune response at the site of infection.

## Introduction

*Staphylococcus aureus* is a common human pathogen that is responsible for a variety of infections, including skin and soft tissue infections, pneumonia, endocarditis, and osteomyelitis. One of the key factors contributing to the virulence of *S. aureus* is its ability to form biofilms, which are communities of bacteria that adhere to a surface and to each other embedded in a self-produced polymeric extracellular matrix^1^. Biofilms provide a protective environment for bacteria, allowing them to evade the host immune response and persist in the host, often resulting in recurrent or chronic infections^2^. Recent studies have demonstrated that biofilms are involved in modulating the host immune response, suggesting that biofilms components play a key role in *S. aureus* immune evasion^1^. Poly-*N*-acetylglucosamine (PNAG), an acetylated polysaccharide, is a common matrix product conferring adhesion in staphylococcal biofilms^3^. Studies in animal models have shown that PNAG-deficient *S. aureus* strains are less virulent than wild-type strains, suggesting that PNAG is an important virulence factor in *S. aureus*^3^.

Pattern recognition receptors (PRRs) are a group of molecules that recognize microbial-associated molecular patterns (MAMPs), which are conserved surface molecules of pathogens^4,5^. There are three main types of PRRs: toll-like receptors (TLRs), NOD-like receptors (NLRs), and C-type lectin receptors (CLRs). TLRs recognize MAMPs from extracellular or phagocytosed bacteria, NLRs recognize bacteria that have penetrated the cell interior, and CLRs recognize carbohydrate-containing PAMPs^6^. Dectin-1 is a CLR that recognizes carbohydrate structures, such as mannans, chitin, and glucans. These carbohydrates are usually found on the surface of fungi^7,8^ but can also be present in some pathogenic bacterial species, such as *Escherichia coli* (type 1 fimbria)^9^ or *Streptococcus mutans* (extracellular glucans)^10^. *S. aureus* does not possess mannans, chitin, or glucans but instead has peptidoglycan (PGN) and teichoic acids, which stabilize its cell wall and are crucial for its pathogenicity and interactions with the host. It is not yet fully understood which receptors of immune cells can recognize PNAG, although there is evidence suggesting that TLR2 and Dectin-1 may play a role in this process^11^. In our previous study, we observed an increase in the secretion of different cytokines and chemokines upon challenge of peripheral blood mononuclear cells (PBMCs) with supernatants of *S. aureus* biofilms. PNAG, as the main extracellular component of the biofilm matrix of *S. aureus*, is likely also present in the supernatants of the biofilms, whereas teichoic acids, which are anchored to the cell wall, do not play a significant role in the composition of the biofilms matrix^12^.

Dectin-1 is expressed in monocytes/macrophages, dendritic cells (DC), and neutrophils^13^. When the respective ligand binds to Dectin-1, it triggers a signaling cascade leading to the activation of various effector functions of immune cells. This signaling cascade involves the phosphorylation of the Dectin-1 receptor by kinases of the Src family, resulting in dimerization of the receptor and the recruitment of the spleen tyrosine kinase (Syk). Syk then activates a number of other signaling molecules, including NF-κB, which is a transcription factor that regulates the expression of genes involved in the immune response, such as chemokines of the CXCL10 subfamily^14,15^. The CXCL10 chemokine subfamily consists of CXCL9, CXCL10, and CXCL11, which belong to the ELR-negative motif (Glu-Leu-Arg) CXC-chemokine superfamily that signals through the CXCR3 receptor. This CXCL9, -10, and -11/CXCR3 axis mainly regulates immune cell chemotaxis through a feedforward signaling loop, differentiation, and activation^16^. These chemokines have also been shown to exhibit antimicrobial activity against different bacteria^17^.

Host chemokines can bind to the cell wall and membrane of *S. aureus*, which in turn stimulates the bacteria to release staphylococcal protein A (SpA)^18^, a virulence factor with adhesive properties that binds to the Fc region of a variety of immunoglobulins. Its binding to antibodies helps the bacterium evade opsonization and phagocytosis by immune cells, thus increasing its survival within the host^19^. One recent study revealed that SpA plays a role in biofilm-associated infections, with reduced biofilm ability of SpA-mutants observed both in vitro and in vivo in a mouse catheter model^20^. Additionally, by promoting CXCL10 secretion, SpA may exacerbate the inflammatory response, potentially leading to tissue damage^18,21^. Recent evidence suggests that *S. aureus* can exploit this response to evade the host immune response by stimulating the secretion of host chemokines^18^. More research is needed to fully understand the role of staphylococcal biofilms in immune evasion, and the stimulatory effect of biofilms matrix components on CXCL10-family chemokine secretion has not been fully explored.

The focus of this study was to investigate the stimulatory effect of PNAG on CXCL-chemokine secretion from immune cells and its connection to the Dectin-1 receptor. Understanding the mechanisms by which PNAG modulates the host immune response will facilitate the development of new therapeutic strategies to combat *S. aureus* infections by targeting the biofilms.

## Results

### Proteome analysis of PBMCs challenged with biofilms

To investigate the interaction of circulating immune cells with biofilms structures, we utilized mass spectrometry coupled with liquid chromatography (LC‒MS/MS). This approach allowed us to evaluate secretome alterations in PBMCs exposed to live plankton or biofilms of *S. aureus*.

**Fig. 1:**
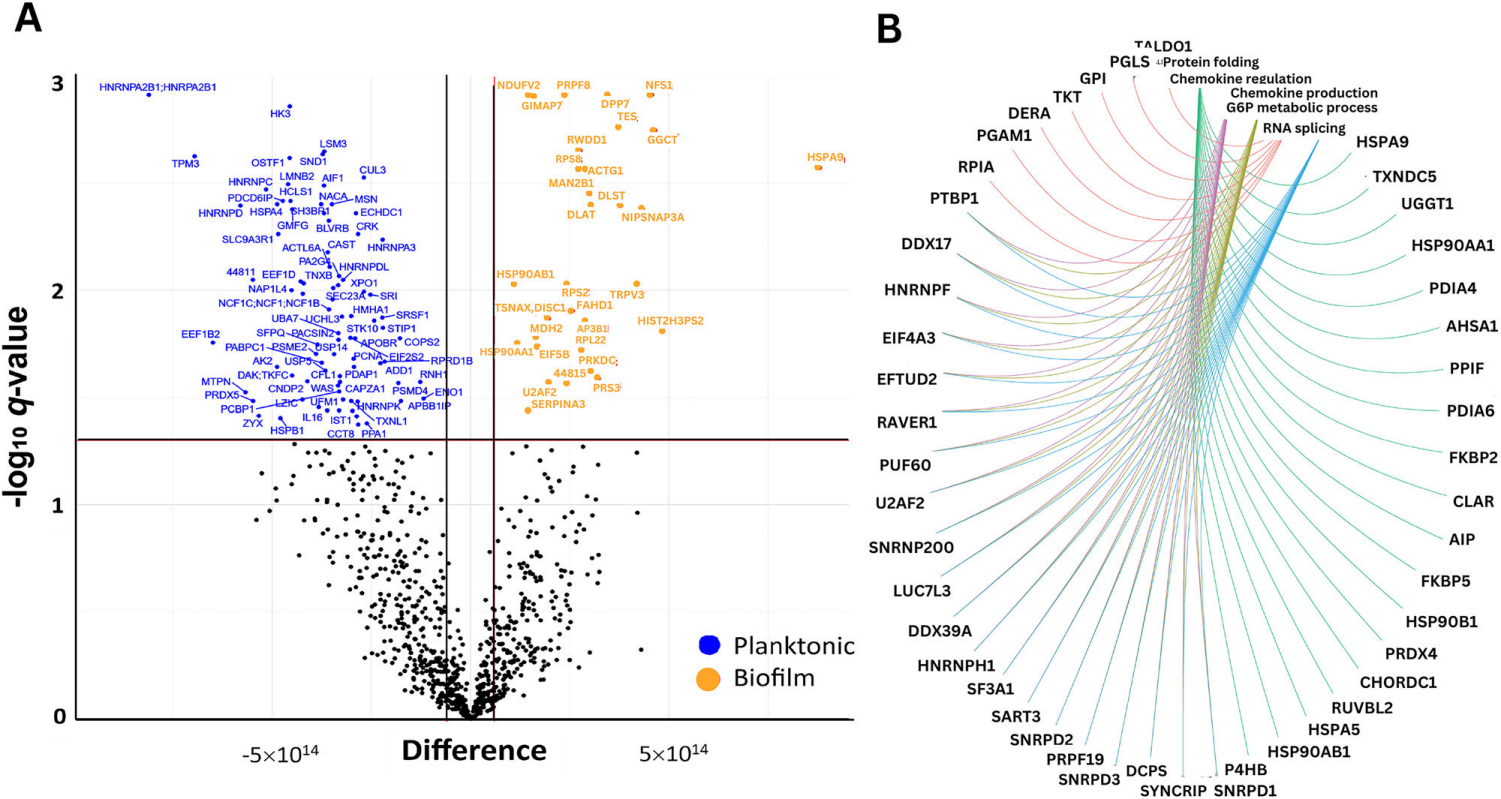
Differential secreted protein analysis and network interactions in Biofilm exposed PBMCs. **A** Volcano plot (-logq versus logFC) showing the difference in the abundance of secreted proteins in PBMCs exposed to biofilms (right). Proteins with a twofold higher (logFC > 1) or lower (logFC < -1) abundance (indicated by the vertical black lines) and BH-corrected p values < 0.05 (indicated by the horizontal red lines) are highlighted (blue and orange, respectively). The significantly differentially abundant proteins were further explored for GO terms and KEGG pathway analysis and visualized. **B** Protein‒protein interaction (PPI) network of proteins differentially secreted by PBMCs upon exposure to biofilms (right). The gene-concept network depicts the linkages of genes and biological concepts (e.g., GO terms or KEGG pathways) as a network. The gene-concept network was constructed to visualize the most relevant nodes engaging in hubs. Edges confidence correlates with the strength of the PPI.

As we reported in our previous study, we identified 1,666 ± 162 differentially expressed proteins (DEPs) in the biofilms-challenged group and 1,305 ± 227 in the untreated control group^22^. Notably, this analysis considered only proteins with a molecular weight greater than 10 kDa because of the filtration step. Thus, chemokines, such as those in the CXCL10 subfamily, which are between 8 and 10 kDa, were not captured. On the basis of the Benjamin‒Hochberg adjusted p value (known as *q* value), proteins with a q value < 0.05 and an abundance fold change (FC) > 2 compared with those in the control groups were identified as having significantly different abundances (Fig. 1A). In PBMCs challenged with biofilms, 321 proteins with significantly higher or lower abundances in cell culture supernatants were identified (for a detailed list of the proteins, see the Supplementary Material, Table S1).

Following enrichment analysis^23^ of DEPs in the biofilms-challenged samples, 44 edges were visualized using a gene concept network plot. The gene-concept network depicts the linkages of genes and biological concepts (e.g., Gene Ontology (GO) terms or KEGG pathways) as a network^24^. For the secreted proteins associated with biofilms challenge in PBMCs (Fig. 1B), several highly connected protein nodes (hubs) were identified. Among these hubs, two were significantly associated with the biological process of chemokine production regulation and neutrophil chemotaxis, as indicated by a significant q value for the corresponding GO term.

### Chemokine secretion of PBMCs challenged with *S. aureus* biofilms

**Fig. 2:**
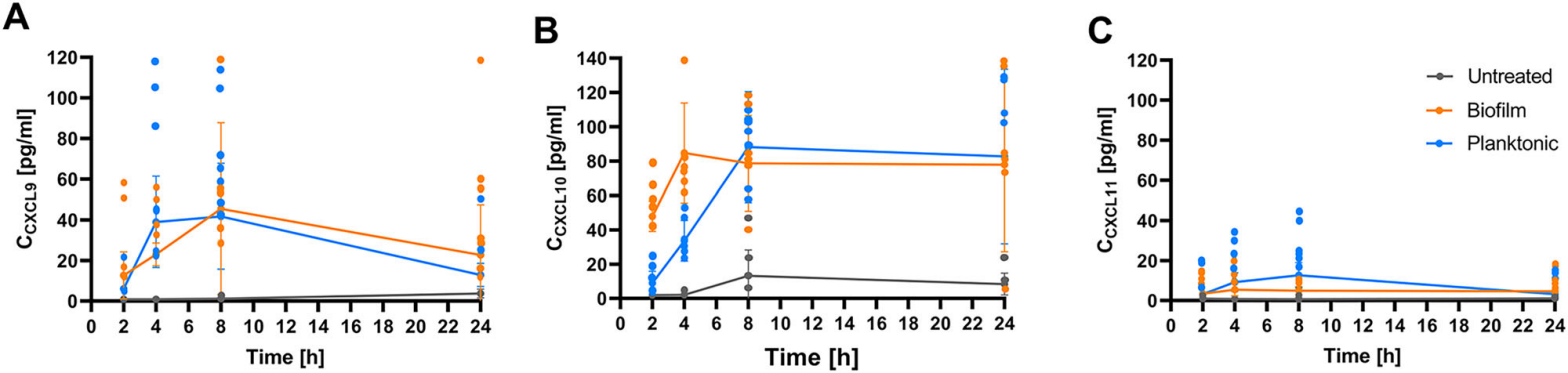
Time-dependent increase in CXC10-like chemokine secretion ratio between PBMCs exposed to planktonic *S. aureus* or the corresponding 24-h-old biofilms and untreated controls. **A** CXCL9, **B** CXCL10 and **C** CXCL11. The values are presented as the means and standard errors of the means (SEMs) of 6 independent experiments.

CXCL10-like cytokines have been previously shown to directly bind to the *S. aureus* cell, with CXCL-9 binding to the cell membrane and CXCL10 to both the cell membrane and the cell wall^18^. We observed the chemotaxis pattern in the proteomics experiment, while we could not capture chemokines such as those in the CXCL10 subfamily, which are smaller than 10 kDa. Therefore, we investigated whether the secretion of CXCL10 subfamily chemokines of PBMC was similarly induced by planktonic *S. aureus* bacteria and their biofilms. The PBMCs were challenged for 24 h with planktonic *S. aureus* suspensions or with one-day-old *S. aureus* biofilms. The time-dependent secretion of the chemokines CXCL9, CXCL10, and CXCL11 was assessed at 2, 4, 8, and 24 h using a bead-based multiplex flow cytometry assay and compared to that of unchallenged PBMCs cultured under the same conditions (control group) (Fig. 2).

In contrast to CXCL9 and CXCL11, which were secreted at very low levels by untreated PBMCs, CXCL10 secretion increased after 8 h and remained stable until 24 h at a level of 72.26 ± 16.57 pg/mL (Fig. 2). When exposed to *S. aureus*, independent of the planktonic or biofilms stage, the secretion of all three chemokines increased. Considering the FC in secretion under stimulation compared with that in untreated PBMCs, both biofilms and planktonic *S. aureus* resulted in similar increases in CXCL9 levels (Fig. 2A), whereas CXCL11 was more strongly stimulated by planktonic bacteria within the first 8 h (Fig. 2C). CXCL10 secretion was greater in the first few hours of stimulation by the biofilms compared to stimulation by the planktonic bacteria, but due to the stimulus-independent increase in the CXCL10 levels in the untreated controls, the stimulus-dependent CXCL10 levels decreased and evened out (Fig. 2B).

These results indicate that the secretion of CXCL10 is stimulated relatively strongly by *S. aureus* biofilms, although the effect cannot be easily differentiated from the stress caused by planktonic *S. aureus*, which also triggers the secretion of chemokines. Nevertheless, we next asked whether a specific component of the biofilms plays a role in this effect. Since PNAG is one of the most important biofilms matrix components, we determined how this polysaccharide compound impacts the secretion of CXCL10 chemokine family members in comparison to other *S. aureus* virulence factors such as SpA, the most abundant and highly conserved virulence factor o*f S. aureus*, which is a cell wall-associated protein (but can also be secreted) that binds to immunoglobulins^25^.

### Effects of PNAG on chemokine secretion in PBMCs

Despite chemical differences among cell wall carbohydrates, such as the varying glycosidic bonds in PGN and the carbohydrate moieties of teichoic acids, they all contain N-acetylglucosamine (GlcNAc), which also forms the homopolymer PNAG. To assess whether PNAG, a component of the biofilms matrix, specifically influences chemokine secretion, we measured CXCL10 levels secreted by PBMCs following a 24-hour exposure to PNAG or the *S. aureus* virulence protein-A. These results were then compared to a control group of unchallenged PBMCs cultured under identical conditions.

**Fig. 3:**
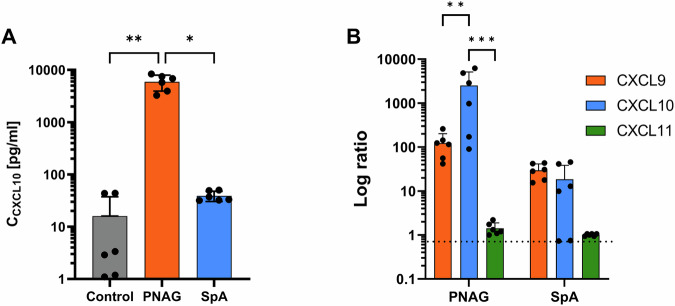
Effects of PNAG and SpA on the secretion of CXCL10. **A** by PBMCs versus the untreated control. **B** Increase in chemokine secretion as a percentage of treated and untreated PBMCs (dashed line). The values are presented as the means and standard deviations (SDs) of six independent experiments. Asterisks indicate significant differences as *p* values * ≤ 0.05 ** ≤ 0.01 and *** ≤ 0.001. *Abbreviations:* PNAG: poly N-acetylglucosamine, SpA: S. aureus virulence protein A.

The CXCL10 level was visibly increased by both compounds, but significance was only achieved when the PBMCs were treated with PNAG but not with SpA (i.e., PNAG in comparison with the untreated control (Fig. 3A) or compared with the SpA-challenged condition). We also examined the secretion levels of the other CXCL10 family members, CXCL9 and CXCL11, in PBMCs treated with PNAG or SpA and compared them to those in unchallenged PBMCs cultured under the same conditions. While both CXCL9 and CXCL11 secretion levels were increased in PBMCs under these treatments, significant differences were observed in the PNAG-treated group compared with the untreated control group (data not shown). Although CXCL11 secretion was significantly greater than that in the SpA-treated group, the secretion level of CXCL11 was generally lower.

A comparison of the ratios of CXCL10 family chemokines between PNAG- or SpA-treated PBMCs and untreated controls (Fig. 3B) confirmed that CXCL10 secretion was preferentially induced by PNAG. To determine whether the observed CXCL10 secretion is specifically induced by PNAG or more generally by carbohydrate-containing components of the bacterial cell, PBMCs were treated for 24 h with staphylococcal PGN, the main polymer of the cell wall. No significant change in CXCL10 levels was observed compared to the untreated control (Supplementary Material, Fig. S2). This suggests that PNAG is the driver of the observed CXCL10 induction.

These results indicate that the matrix-derived product PNAG significantly affects immune cell chemotaxis by increasing the production of the chemokine CXCL10. CXCL10 was stimulated with a clear predominance by PNAG. CXCL10, which is a ligand of the CXCR3 axis, primarily regulates immune cell extravasation, differentiation, and activation, promoting the recruitment of different immune cells, such as cytotoxic lymphocytes, natural killer cells and mucosal-associated invariant cells^26^.

### Transcriptome analysis of PBMCs challenged with PNAG

Three possible signaling pathways can be controlled by MAMPs in immune cells: i) TLRs, ii) NLRs and iii) CLRs. CLRs specialize in recognizing polysaccharide residues or motifs, such as *β*-glucans, which are components of the fungal cell wall^27^. *β*-glucan in fungi is a linear polysaccharide of glucose, whereas PNAG is a polysaccharide composed of polymeric linked N-acetylglucosamine that can be produced in some bacteria, such as *S. aureus*^28,29^. We aimed to investigate whether PNAG, similar to β-glucan in fungi, can stimulate the expression of this receptor and related signaling processes.

**Fig. 4:**
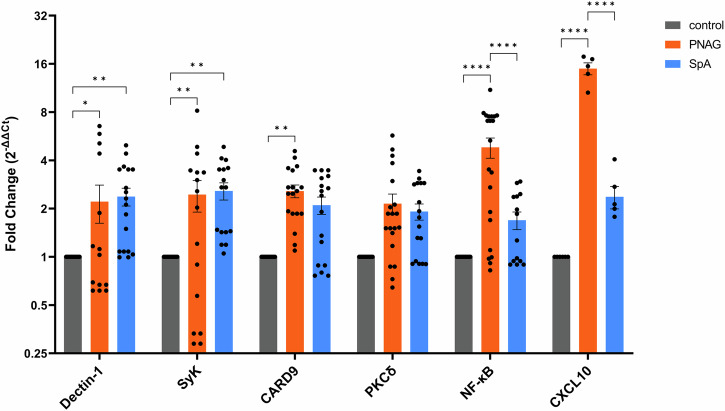
Relative expression of the selected genes (X-axis) in the PBMCs treated with PNAG or SpA expressed as 2^-ΔΔCt^ normalized to the reference gene (18S rRNA). The values are presented as the means and standard errors (SE) of 6 independent experiments. Significance is indicated as follows: * *p* < 0.05, ** *p* ≤ 0.01, **** *p* ≤ 0.01. *Abbreviations:* PNAG: poly N-acetylglucosamine, SpA: S. aureus virulence protein A.

PBMC were incubated for 24 h in the presence of PNAG, and the mRNAs of six genes encoding Dectin-1, C-type lectin domain family 7 member A, Syk as a signaling adapter, caspase recruitment domain-containing protein 9 (CARD9) signaling adapter mediating the inflammatory response for C-lectin pattern recognition, protein kinase C-delta (PKCδ), nuclear factor kappa-light-chain-enhancer of activated B cells (NF-κB), and CXCL10 were transcribed into complementary DNA (cDNA) using reverse transcription. Quantification via the TaqMan qPCR method was normalized to the expression of the 18S rRNA gene^30^. The expression level of the 18S rRNA gene ranged from 7.91 ± 0.22 to 8.92 ± 0.14 cycles (means and standard deviations (SDs)), with no significant difference between the groups (Supplementary Material, Fig. S1), confirming its suitability as a reference gene in this experimental setup. The mean ΔC_t_ value of the control group was subtracted from all individual values, including the control group values, to visualize the variation within this group. Additionally, the 2^-ΔΔCt^ values of all the groups were calculated to illustrate the expression patterns of all the target genes (Fig. 4).

TaqMan qPCR confirmed the results observed for cytokine protein secretion, revealing a greater than 10-fold increase in the mRNA expression of CXCL10 after PNAG treatment compared with that in the SpA or control conditions. When the effects of PNAG and SpA on the expression of the Dectin-1-Syk-CARD9 signaling pathway were compared, a specific and large increase (FC) of 4.81 ± 0.69) in NF-κB expression by PNAG was detected upon PNAG challenge. Compared with the control, PNAG also significantly elevated the expression of Dectin-1 (FC 2.12 ± 0.53), CARD9 (FC 2.83 ± 0.55), and Syk (FC 2.46 ± 0.54). PKCδ expression remained unchanged compared with that in the untreated control.

Given the observed PNAG-mediated signaling, we assessed whether *S. aureus* PGN could also activate this pathway. PBMCs were treated with PGN for 24 h and the same targeted transcriptomics was measured. Transcriptomic analysis showed a significant upregulation of NF-κB (FC 2.1 ± 0.69) but no significant changes in the other four target mRNAs examined (Supplementary Material, Fig. S3A). Pretreatment of PBMCs with an anti Dectin-1 blocking antibody did not abrogate PGN induced NF-κB upregulation (Supplementary Material, Fig. S3B), suggesting that PGN activates NF-κB independently of Dectin-1. We further investigated whether PGN induced NF-κB activation affects CXCL10 mRNA expression. We observed that PGN treatment did not induce CXCL10 expression (Supplementary Material, Fig. S3C), indicating that the PGN activated NF-κB does not converge on CXCL10 expression.

NF-κB is a master regulator of the inflammatory response to pathogens, and its expression can be stimulated through a variety of pathways involving diverse pathogen recognition receptors and chemokines, including the Dectin-1-Syk- pathway^31^. Thus, these results suggest that the major *staphylococcal* biofilms matrix PNAG stimulates NF-κB expression via this pathway. Moreover, the expression of the essential signaling components of the Dectin-1-Syk-CARD9 cascade was also increased in the presence of PNAG. Dectin-1 expression is activated by various cytokines and chemokines^11^, but how this process is related to PNAG is still unclear^32^. As shown above, PNAG has a significant effect on the proteome of PBMCs and influences the expression of chemokines, thereby modulating immune chemotaxis.

### Chemokine secretion in PBMCs after dectin-1 blockade or SyK inhibition

**Fig. 5:**
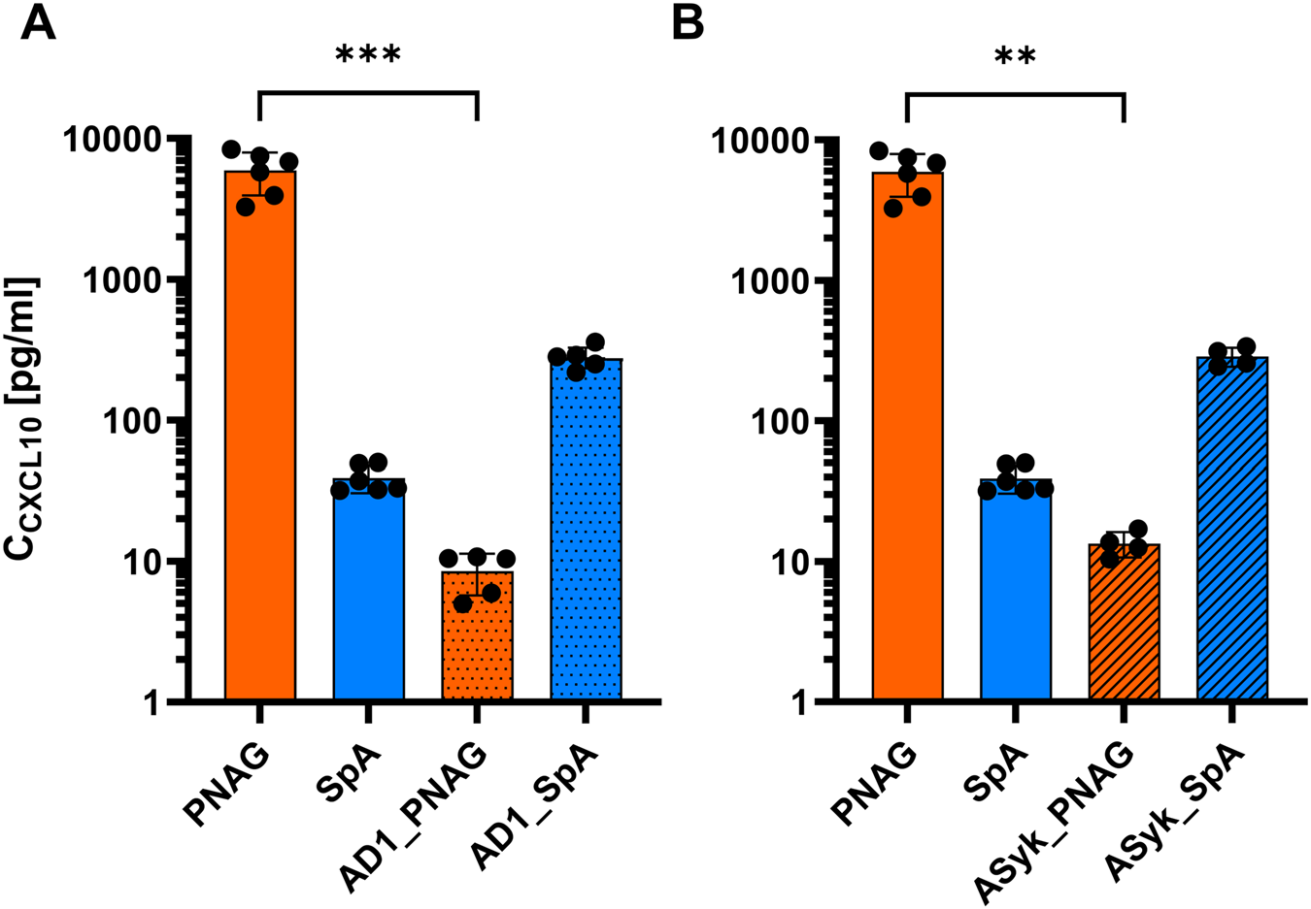
Effects of Dectin-1 blockade and Syk inhibition on CXCL10 secretion by PBMCs. **A** Secretion of CXCL10 with/without antibody blockade of Dectin-1 in PBMCs treated with PNAG or SpA. **B** Levels of secreted CXCL10 in PBMCs with/without inhibition of the protein tyrosine kinase SyK. The values are presented as the means and standard deviations (SDs) of three independent experiments. Asterisks indicate significant differences as p values * ≤0.05 ** ≤0.01 and *** ≤0.001. *Abbreviations:* AD1_PNAG or SpA: Antibody-blocked Dectin-1 PBMCs treated with PNAG or SpA, ASyK_: SyK inhibited PBMCs treated with PNAG or SpA, PNAG or SpA: PBMCs treated with PNAG or SpA.

We blocked Dectin-1 with a blocking antibody and then measured the secretion levels of the chemokine CXCL10 by PBMCs in response to a 24 h treatment with the *S. aureus* biofilms component PNAG or SpA and compared these levels with those of unchallenged PBMCs cultured under the same conditions. Compared with that in PBMCs without antibody blockade under the same treatment, the CXCL10 level in PBMCs under PNAG treatment significantly decreased after antibody blockade against Dectin-1; however, this difference was not significant for SpA-treated Dectin-1-blocked PBMCs compared with PBMCs without blockade antibody (Fig. 5A). The level of CXCL10 secretion was altered in unchallenged PBMCs after treatment with an antibody blockade against Dectin-1, but the difference was not significant (Supplementary Material, Fig. S4A).

Afterward, we inhibited protein tyrosine kinase Syk in PBMCs via piceatannol, followed by a 24-hour challenge with either PNAG or SpA. We then measured the CXCL10 secretion levels in these treated PBMCs and compared them to those in unchallenged PBMCs cultured under the same conditions. The results revealed a significant decrease in CXCL10 secretion in Syk-inhibited PBMCs treated with PNAG compared with that in PBMCs without Syk inhibition under the same treatment (Fig. 5B). The secretion of CXCL10 by PBMCs tended to increase in the SpA-treated group but was not significant. Similar to dectin-1 antibody blockade, the levels of CXCL10 secretion in unchallenged PBMCs after piceatannol inhibition did not change significantly compared to those not treated with piceatannol (Supplementary Material, Fig. S4B).

This finding suggests that Dectin-1 and SyK signaling adapters may play a direct role in the immune chemotaxis induced by the biofilms matrix-derived product PNAG on immune cells during chronic infection. As discussed in the previous section, PNAG predominantly induces the secretion of CXCL10 through the Dectin-1 receptor and its corresponding signaling pathway. We observed that this predominance of CXCL10 is a distinct feature of PNAG, involving Dectin-1 and its corresponding signaling pathway. The CXCL10/CXCR3 axis is involved in regulating immune cell chemotaxis, leading to the infiltration of specific immune cell subtypes, such as T cells, which may aid in the clearance of pathogens.

### Proteome analysis of monocytes treated with PNAG

**Fig. 6:**
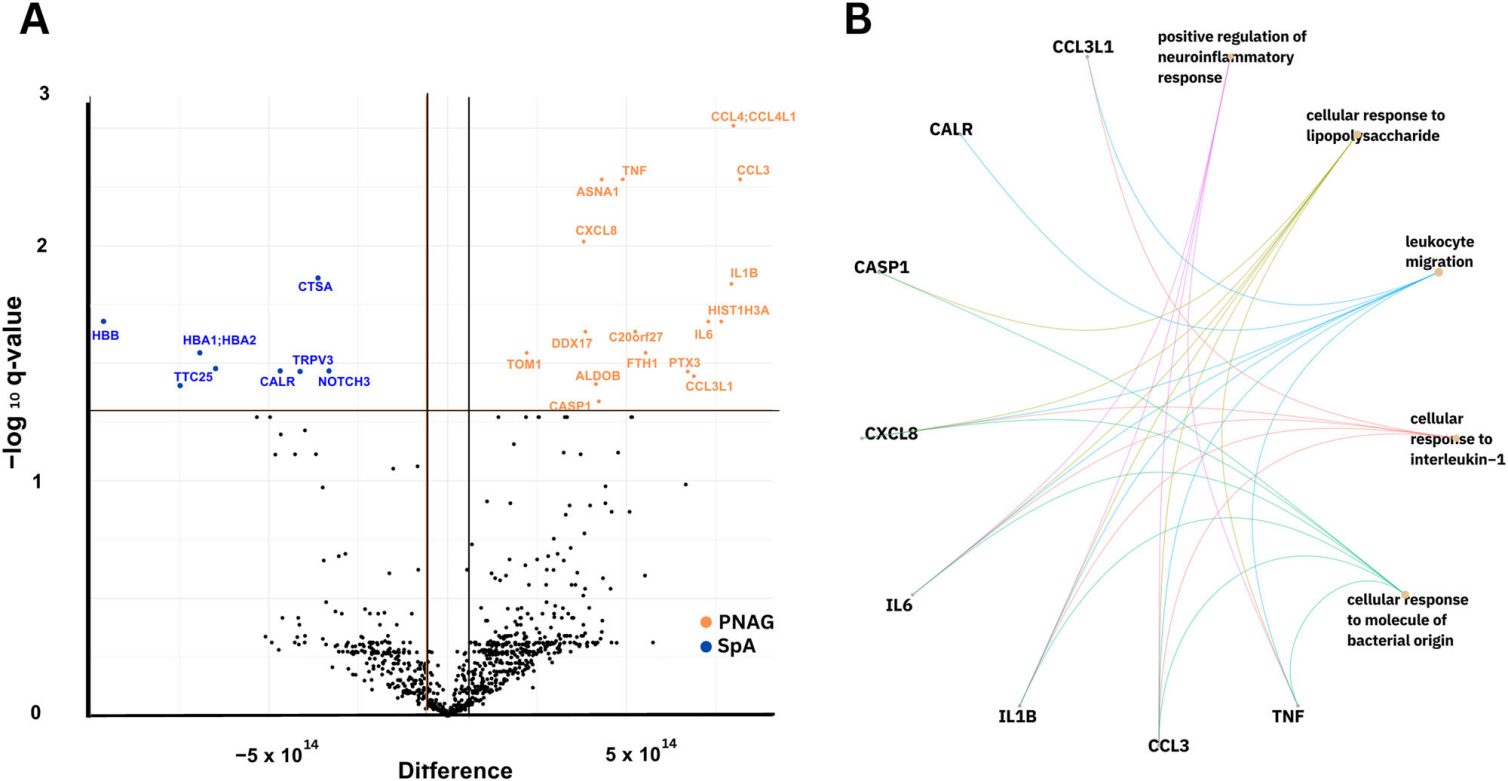
Differentially expressed proteins in monocytes upon PNAG treatment. **A** Volcano plot (-log_*q*_ versus log_FC_) showing the difference in the abundance of secreted proteins in monocytes exposed to PNAG. Proteins with significantly differential secretion expressed as *q* values < 0.05 (indicated by the horizontal red line) and twofold higher (log_FC_ > 1, red) or lower (log_FC_ < -1 blue) secretion _(_indicated by the vertical red lines) are highlighted. **B** Gene-Concept network plot of proteins differentially secreted by monocytes upon exposure to PNAG. The gene-concept network was constructed to visualize the most relevant nodes engaging in hubs. Edges confidence correlates with the strength of the PPI. *Abbreviations:* PNAG: poly N-acetylglucosamine, SpA: S. aureus virulence protein A.

We detected a secretome change in isolated monocytes exposed to PNAG and identified 1,447 ± 207 or SpA 1,353 ± 201 DEPs in comparison with the untreated control group. We further visualized the differential proteomic results between the two treatment groups on the basis of the Benjamini‒Hochberg adjusted *p* value (*q* value < 0.05) and an abundance FC > 2 as a volcano plot (Fig. 6A). In the secretome of monocytes exposed to PNAG or SpA, 24 protein groups were detected with a significant differential abundance, with 16 proteins higher (*N* = 16, red) in the PNAG group and eight higher in the SpA group (for a detailed list of the proteins, see Supplementary Material, Table S2).

The significantly DEPs were further explored by GO term analysis and visualized as a Gene-Concept network (Fig. 6B)^23,24^. A total of 33 edges were identified with two major hubs and many poorly connected nodes under the PNAG challenge. Cluster one was composed of 15 proteins involved in cytokine activity and regulation, leukocyte chemotaxis, and the inflammatory response. The proteins with the most significant and greatest increase in secretion were CCL4, CCL3, CXCL8, tumor necrosis factor *α*, and IL-1β. Within this cluster, three downregulated proteins were also involved: i) tetratricopeptide domain 25 (TTC25), a component of the outer dynein arm-docking complex subunit 4 that is involved in ciliary and flagellar motility, whose ciliary dysfunction is often associated with recurrent respiratory infections^33,34^; ii) NOTCH3, which plays a role in the activation of NF-κB^35^; and iii) calreticulin (CALR), a calcium-binding chaperone involved in the assembly of the endoplasmic reticulum (ER)^36^. The second cluster was related to stress and defense responses but contained primary proteins downregulated in the presence of PNAG (the hemoglobin subunits HBB and HBA and orosomucoid 1 protein (ORM1)) **(**Fig. 6B**)**.

Although our primary focus was on CXCL10-family chemokines, their small size^37^ made them challenging to capture during LC‒MS/MS analysis, possibly due to filtration during sample preparation. Nonetheless, these results confirmed the occurrence of chemotaxis-related changes in the monocyte proteome. This observation suggests that PNAG plays a role in initiating chemotaxis, thereby recruiting immune cells to the site of infection.

### Effect of PNAG on monocyte phenotypes

**Fig. 7:**
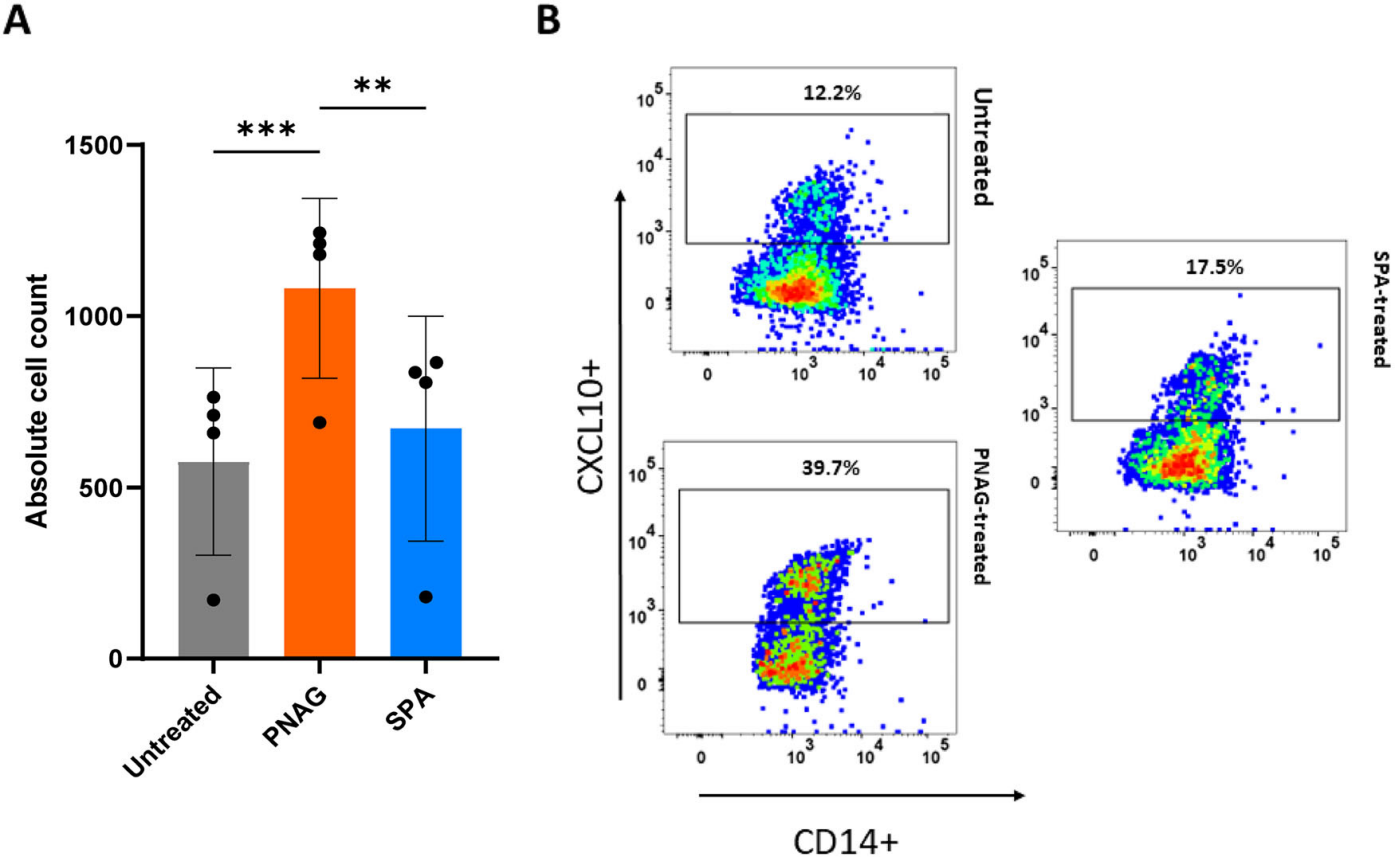
Analysis of monocytes using fluorescence-activated cell sorting for evaluation of CXCL10 and the CD14 surface marker. **A** The absolute cell count of CXCL10^+^ monocytes evaluated under PNAG- or SpA-treated conditions compared with the untreated control. The values are presented as the means and standard deviations (SDs) of three independent experiments. **P ≤ 0.01, ***P ≤ 0.001. **B** Dot plot representing the frequency of specific monocytes treated with PNAG or SpA compared with the untreated control. The *X*-axes correspond to the CD14+ receptor, and the *Y*-axes correspond to the CXCL10 signals. *Abbreviations:* PNAG: poly N-acetylglucosamine, SpA: S. aureus virulence protein A.

We aimed to further investigate how the phenotype of monocytes, the main modulators of the cytokine milieu at the site of infection^38,39^, changes upon interaction with PNAG and SpA. We treated isolated monocytes with PNAG or SpA followed by 24 h of incubation and observed potential changes in monocyte phenotypes via fluorescence-based staining with monoclonal antibodies against CD14 (a cell surface marker) and intracellular CXCL10 **(**Fig. 7**)**.

Compared with that of the untreated control, the number of CXCL10-expressing monocytes remained unchanged after SpA treatment (Fig. 7A), whereas the presence of PNAG led to a significantly increased number of CXCL10-expressing monocytes. Flow cytometric analysis revealed that up to 12.2% of the CD14^+^ CXCL10+ monocytes in the untreated control group and 17.5% of the SpA-treated monocytes were positive, whereas 39.7% of the PNAG-treated monocytes were positive in some experiments (Fig. 7B).

Flow cytometry analysis confirmed that PNAG induces the formation of CD14^+^CXCL10^+^ monocytes that migrate to the site of infection and trigger the innate immune response against *S. aureus*. The specific process through which CD14+CXCL10+ monocytes initiate the innate immune response remains elusive. However, it is possible that this process leads to the activation of TLRs or other PRRs and the subsequent release of proinflammatory cytokines.

### Impact of CXCL10 on the growth and biofilms formation of *S. aureus*

On the basis of these results, we focused on CXCL10 and investigated the effect of CXCL10 on *S. aureus*. We added recombinant CXCL10 to a planktonic suspension of GFP-labeled *S. aureus* and assessed the increase in viable planktonic bacteria and biofilms mass up to 24 h at intervals of 30 min by using a heatable plate reader with orbital shaking (planktonic stage) or confocal laser scanning microscopy (CLSM, biofilms stage). The optical density at 600 nm was assessed for the planktonic bacteria. The biomass of the biofilms was quantified for images with the highest density (just above the bottom of the glass) as the sum of the fluorescence signal intensity (see the Materials and Methods section for details) and as viable bacteria (colony forming units (CFU)/mL) after microdilution and plating on agar.

CXCL10 had a weak inhibitory effect on planktonic growth, as observed for 12 h at 500 nM CXCL10 (Supplementary Material, Fig. S5A). The growth rate of planktonic was not significantly influenced by CXCL10, but the culture reached the stationary phase at a lower OD_600_ than did the lower concentrations and the untreated control. Interestingly, after 24 h, the effect was reversed, and a decrease in the OD_600_ was evident at 250 nM compared with 500 nM CXCL10 (Supplementary Material, Fig. S5B), while a concentration of 100 nM did not significantly change the OD_600_. The increased density observed at higher CXCL10 concentrations likely reflects the development of biofilms with enhanced resistance to stress. This induction of biofilms development by CXCL10 challenge may involve specific signaling pathways leading to increased matrix production or altered bacterial aggregation.

**Fig. 8:**
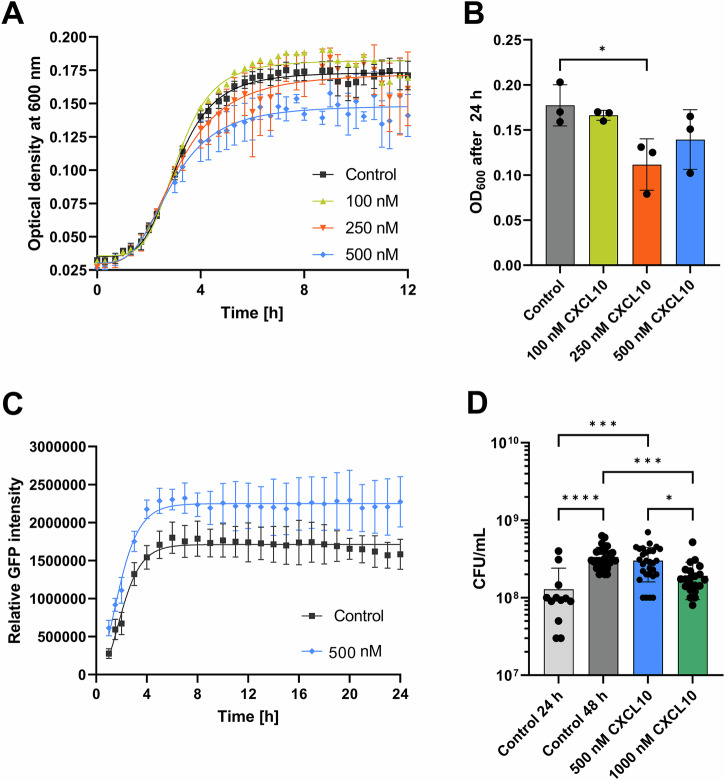
Effects of CXCL10 on the planktonic growth and biofilms formation of *S. aureus.* **A** Growth curves of planktonic *S. aureus* recorded for 12 h at 37 °C in a 96-well plate and nonlinearly fitted. **B** Optical densities of the planktonic cultures after 24 h. **C** Relative GFP intensity of the biofilms layer formed on the glass coverslip over time. **D** Viable *S. aureus* (CFU/mL) fraction of the 24 h mature biofilms before and after treatment for an additional 24 h with different concentrations of CXCL10 or the untreated control for 48 h. Values are presented as the means and standard deviations (SDs) of three independent experiments. Asterisks indicate significant differences as *p* values * ≤ 0.05, *** ≤ 0.001, **** ≤ 0.001.

To assess the influence of CXCL10 on biofilms formation, we quantified the biofilms mass over time using the constitutive GFP-labeled bacteria. We observed that treatment of planktonic bacterial with 500 nM CXCL10 resulted in faster initial attach to the glass surface (Supplementary Material, Fig. S5 A-B) and formation of bacterial aggregates which leading to higher GFP intensities than those in the absence of CXCL10 (Fig. 8C). To prove that the observed effect of CXCL10 is specific we treated the bacteria with heat-inactivated CXCL10 or phosphate-buffered saline (PBS). We found that neither heat-inactivated CXCL10 nor PBS (Supplementary Material, Fig. S5C, D) had a discernible effect on the initial attachment phase of planktonic bacteria to the glass surface. This lack of effect suggests that the previously observed enhancement of early biofilm formation is specifically mediated by the active form of CXCL10. The visualization of biofilm mass clearly increased in the presence of CXCL10 (Supplementary Material, Fig. S5B) compared with that of other conditions within 24 h. In the presence of CXCL10, increased bacterial aggregation was observed after 1 h compared with that of the untreated *S. aureus*. After that point, bacterial aggregation was less clearly recognizable, but the biofilms mass increased visibly in the presence of CXCL10.

We further investigated the effect of CXCL10 on mature biofilm growth by quantifying CFUs after treatment with 500 nM and 1 µM CXCL10 **(**Fig. 8D**)** for 24 h. We increased the CXCL10 concentration because of the greater tolerance of the biofilms to chemical treatments. Prior to treatment, the mature biofilms contained approximately 1.2 × 10^8^ CFU/mL. After further incubation for 24 h (untreated controls), the biofilms reached 3.4 × 10^8^ CFU/mL. The concentration of 500 nM CXCL10 did not affect bacterial count within biofilm. A higher concentration of CXCL10 (1 µM) appears to have a negative effect on bacteria number. This is evident from the lower number of viable bacteria (1.9 CFU/ml) compared to the control and 500 nM CXCL10 (Fig. 8D). However, it remains unclear whether this apparent reduction in viable bacteria at 1 µM CXCL10 was due to direct inactivation of bacterial cells or overall inhibition of biofilm expansion. It is also unclear whether CXCL10 interacts specifically or nonspecifically with biofilm-embedded bacteria or matrix components at higher protein concentrations.

Thus, at relatively high concentrations, CXCL10 has some inhibitory effects on biofilms growth. On the other hand, the presence of CXCL10 can also trigger biofilms formation, which can be an escape strategy of *S. aureus* from host immune cells. However, it cannot be completely ruled out that the observed increase in the GFP signal could be a bias from increased metabolic activity due to CXCL10 challenge.

## Discussion

Biofilms-associated infections, particularly those caused by *S. aureus*, pose a major burden on the healthcare system. It is therefore important to understand the interplay between biofilms and immune chemotaxis and to develop new therapeutic approaches that specifically target biofilms or can influence their immune response to benefit the host. To do this, however, the structures that are particularly suitable for targeted therapy of infections prone to becoming chronic must be identified.

Immune cells such as monocytes secrete chemokines in response to bacteria, which can trigger the release of various virulence factors or lead bacteria to other escape mechanisms, such as intracellular hiding or biofilms formation. Increased secretion of CXCL10 has been observed in response to various infections, including those caused by *S. aureus*^39^. Host chemokines, particularly CXCL10, have been shown to bind directly to *S. aureus*, inducing the secretion of the virulence factor SpA. This has been proposed as a potential immune evasion mechanism, in which the pathogen exploits a host defense factor to release a virulence factor^18^. The biofilms extracellular matrix acts as a shield to protect the bacterial community against the immune response and antibiotic treatment. The major biofilms matrix compound in *S. aureus* is the polysaccharide PNAG. Here, we demonstrated that PNAG stimulates the secretion of CXCL10 from PBMCs and monocytes and that, in turn, CXCL10 may promote the maturation of *S. aureus* biofilms at an early stage. This observation provides some insights into how *S. aureus* benefits from host chemokine secretion. However, molecular evaluation is needed to verify these findings in greater detail and to understand the cellular processes in *S. aureus* due to the presence of CXCL10. The specificity of the role of PNAG in immune activation in *S. aureus* compared with other bacterial species remains an open question that also needs to be addressed in future research.

The production of CXCL10-like chemokines by PBMCs or monocytes due to *S. aureus* infection indicates targeted infiltration of further specific immune cells to the site of infection^40^. Our results showed that the CXCL10/CXCR3 chemotaxis pathway is predominantly initiated by biofilms-specific PNAG, and particularly CXCL10 is a distinct feature of the interaction of PNAG with Dectin-1. This finding is in line with our previous findings, as a significantly stronger and more aggressive immune response against inert biofilms is required^22,41^. However, it remains unclear whether this response ultimately results in a reduction in biofilms mass in vivo.

On the other hand, PNAG is most likely recognized by Dectin-1, which is a PRR for a variety of β-linked polysaccharides^42^. When stimulated with glucan, a Dectin-1 agonist and a component of fungi, Dectin-1-Syk-CARD9 signaling has been shown to induce dendritic cell maturation, which in turn promotes the chemotaxis and differentiation of CD4+ IL-17-producing effector T cells (TH-17 or TH-1 cells) in vitro^31,43^ and can thus stimulate the long-term memory of immune cells. Our findings suggest that PNAG, as a biofilms matrix component of *S. aureus*, may modulate immune chemotaxis through the Dectin-1-Syk-CARD9 pathway, potentially influencing the long-term memory and chronification associated with biofilms formation by *S. aureus*. This represents a significant step forward in understanding the mechanisms by which *S. aureus* biofilms interact with the host immune system. However, whether PNAG specifically contributes to the modulation of immune chemotaxis and, subsequently, long-term immune memory and its implications for chronic infection and biofilms formation by *S. aureus* remain open questions. Future researchers exploring potential therapeutic interventions could focus on this pathway to provide novel strategies for preventing or treating chronic biofilms-associated infections.

### LIMITATIONS OF THE STUDY

While this study provides valuable insights into the interaction between *S. aureus* biofilms and immune chemotaxis, several limitations should be acknowledged. The primary limitation of this study lies in its in vitro design. While the use of PBMC offers advantages in understanding immune responses, it does not fully capture the complexity of in vivo interactions. We did not encompass neutrophils, which play a significant role in innate immunity against pathogens. Although this was a deliberate choice to address our primary research question, we recognize the importance of neutrophils and intend to explore the effects of biofilm complex carbohydrates such as PNAG on neutrophil responses in future investigations. The absence of all immune cells and plasma components in the culture system could impact the translatability of these findings to in vivo scenarios.

Another limitation is that this study focused on PNAG as a biofilm matrix component, but biofilms are composed of various molecules and structures. The role of other biofilms constituents or complex carbohydrates in immune modulation remains unexplored in this study.

In addition, the effects of CXCL10 on bacterial growth and biofilms were determined with CXCL10 concentrations higher than those described for human samples; therefore, the effects may not be clinically relevant.

The natural environment of *S. aureus* is the human body, which has a physiological salt concentration. Therefore, our experiments were conducted under these conditions to reflect the relevant biological context. However, it may indeed be interesting to investigate how lower or higher NaCl concentrations affect this interaction. Previous studies suggest that lower salt concentrations can enhance *S. aureus* virulence by promoting biofilms formation via the *ica* operon, which encodes the machinery for PNAG production^44^. Therefore, investigating the role of salt concentration in S. aureus infections and its interaction with host factors like CXCL10 could clarify the mechanisms behind bacterial persistence and immune evasion.

Finally, we used a methicillin-resistant *S. aureus* (MRSA) strain in this study due to results from a preliminary experiment in which we observed total lysis of immune cells upon exposure to methicillin-sensitive strains (MSSAs) (unpublished). MSSA tends to exhibit a more aggressive response to immune cells than does MRSA^45^, which may experience fitness costs associated with the additional expression of resistance genes, such as *mecA*, and the associated altered PGN structure^46^. Thus, we cannot exclude the possibility that MSSA biofilms may have different effects on chemokine responses. This point should be addressed in future research.

In conclusion, while this study provides significant insights into the interaction between *S. aureus* biofilms and immune chemotaxis, its limitations highlight the complexity of immune responses in in vivo scenarios. Future studies should include a combination of in vitro and in vivo studies with a focus on specific cell types, patient cohorts, and biofilms constituents for a more comprehensive understanding of *S. aureus*-related immune modulation.

## Resource availability

### Lead contact

Further information and requests for resources and reagents should be directed to and will be fulfilled by the lead contact, Reza Gheitasi (reza.gheitasi@ukbonn.de).

## Methods

### Storage and culturing of bacterial strains

The reference strain *S. aureus* ATCC 43300 was purchased from American Type Culture Collection (Manassas, USA). We chose to use a laboratory standard ATCC strain to ensure comparability for future studies that may wish to compare their results with ours. The constitutively GFP-labeled USA300GFPBas strain used for biofilms imaging was kindly provided by Bas G. J. Surewaard at Snyder Institute for Chronic Diseases, Department of Physiology and Pharmacology, University of Calgary, Calgary, Canada. Stock cultures were established by cultivating bacteria in tryptic soy broth (TSB) (Merck KGaA, Darmstadt, Germany) overnight. The bacterial suspensions were then aliquoted and stored in the same medium supplemented with 10% glycerin at −80 °C. For the experiments, the bacteria were streaked from the stocks on blood agar plates (Merck KGaA) and incubated overnight at 37 °C. Fresh TSB cultures were prepared by resolving a single colony in 10 mL of TSB and incubating overnight with shaking (150 rpm) at 37 °C. From these overnight cultures, a volume of 0.5 mL was centrifuged at 3500 × g at RT for 5 min, and the pellet was resuspended and adjusted to an optical density of 0.07 at 600 nm (OD_600_) in enriched RPMI medium (containing 10% FBS and 2 µM L-glutamine). In our previous study, we demonstrated that *S. aureus* forms suitable biofilms in this cell medium^22^F. The CFUs per mL in the inoculum suspensions were confirmed by log-microdilution and colony counting on TSB agar plates after incubation overnight at 37 °C. For biofilms formation, 200 µL of the bacterial suspensions were added per well into a 96-well flat-bottom plate (Greiner Bio-One GmbH, Frickenhausen, Germany) in triplicate and incubated for 24 h at 37 °C without shaking in humidified and 5% CO_2_ conditions. The biofilms were gently washed three times with prewarmed PBS (Carl Roth, Karlsruhe, Germany) to remove free planktonic cells and subsequently exposed to different treatments as described below.

### PBMCs and isolation of monocytes

Peripheral blood from anonymized healthy volunteers was purchased from the Institut für Klinische Transfusionsmedizin Jena GmbH (Jena, Germany). To isolate PBMC, the blood collected in 10 mL EDTA K3 tubes (S-Monovette®, Nümbrecht, Germany) was diluted 1:1 with PBS in a 50 mL tube (Falcon Corning, NY, USA). The mixture was layered with Lymphouprep^TM^ density gradient medium (Stemcell Technologies Inc., Vancouver, Canada) and centrifuged at 1200 × g for 15 min at room temperature (RT) according to the manufacturer’s protocol. After centrifugation, the PBMC layer was transferred into a new conical tube, washed three times with 5 mL of PBS containing 2% heat-inactivated fetal bovine serum (FBS), and finally resuspended in RPMI medium (if needed, supplemented with 10% FBS and 2 mM L-glutamine) (all Thermo Fisher Scientific, Waltham, USA). The viable cells were counted after they were stained with 0.4% trypan blue solution (Thermo Fisher Scientific) using a Countess 3 device (Thermo Fisher Scientific).

To isolate the monocytes, the PBMC pellet was resuspended in 4 mL of MojoSort buffer (BioLegend, San Diego, USA) in polypropylene tubes according to the manufacturer’s instructions. The suspension was centrifuged at 300 × g for 5 min, the pellet was resuspended in the recommended volume of MojoSort buffer, and 5 µL of Fc receptor blocking solution (Human TruStain FcX, BioLegend, San Diego, USA) was added. This suspension was incubated at RT for 10 min, and subsequently, 10 µL of a biotin-antibody cocktail was added, and the mixture was incubated on ice for 15 min. Finally, 10 µL of streptavidin nanobeads (BioLegend, San Diego, USA) was added to the cells, followed by incubation on ice for 15 min. The cells were washed with 4 mL of MojoSort buffer and centrifuged at 300 × g for 5 min. The resulting supernatant was discarded, and the pellet was resuspended in 2.5 mL of MojoSort buffer. The tube was placed in a magnet device (BioLegend, San Diego, USA) for 5 min, after which the mixture was removed, and the mixture containing the isolated monocytes was collected.

### Challenge of PBMCs and monocytes with *S. aureus*, PNAG, SpA and PGN

The PBMCs were adjusted to 1 × 10^6^ per 200 µL of enriched RPMI medium., The 200 µL PBMC aliquots were directly applied onto 24 h-old *S. aureus* biofilms (~1 × 10^7^ CFU/well). For the experiments with planktonic *S. aureus*, the PBMC aliquots were pelleted at 350 × g for 5 min and resuspended in 100 µL, which was mixed with 100 µL of a 1 × 10^7^ CFU/mL suspension. These cocultures were incubated at 37 °C and 5% CO_2_ for 24 h. PBMCs alone were cultured under identical conditions and served as untreated controls. The supernatants were collected via centrifugation at 500 × g for 5 min at RT, sterile filtered (0.22 µm pore size) using a syringe filter (B. Braun AG, Melsungen, Germany), aliquoted, and stored at -80 °C until further analysis (e.g., chemokine determination).

Isolated PBMCs and monocytes were resuspended in enriched RPMI (1 × 10^6^ cells/well), and 1 ml of cell suspension was added to a 24-well plate. The cells were treated with or without 500 ng/10^6^ antibody against human Dectin-1 (InvivoGen, Toulouse, France) or 50 µM piceannnol (Merck KGaA) for 30 min and then treated with PNAG (100 μg/mL), SpA (10 μg/mL) (all Thermo Fisher Scientific), or PGN (100 μg/mL) (Sigma Aldrich) for 24 h at 37 °C in 5% CO_2_. As controls, untreated PBMCs and monocytes were cultured in the same manner. After incubation, the supernatants were collected by centrifugation at 350 × g for 5 min at RT and sterile filtered (0.22 µm pore size). The filtered supernatants were aliquoted and stored at −80 °C until further chemokine determination. The monocyte pellets were resuspended in PBS and used for cell marker evaluation (see below).

### Challenge of *S. aureus* with CXCL10

The lyophilized recombinant human CXCL10/IP10 protein (Abcam, Cambridge, UK) was prepared according to the manufacturer’s instructions. The bacterial mixture with an OD_600_ of 0.07 was adjusted in enriched RPMI medium (containing 103.4 mM NaCl) supplemented with 100, 250, or 500 nM CXCL10. As a control untreated bacterium was used, and as additional control for random effect of active form of CXCL10 protein, a 500 nM CXCL10 heat inactivated at 95 °C for 30 min or with PBS was used. Then, 200 µl of the fresh bacterial suspension was placed in a 96-well plate (Greiner Bio-One GmbH). The bacterial growth rate under different conditions was subsequently monitored using an Infinite 200 PRO plate reader (Tecan Trading AG, Männedorf, Switzerland). Moreover, to assess the effect of CXCL10 on biofilms growth activity, 200 µl of enriched RPMI containing 500 nM or 1 µM CXCL10 was added to a 24 h-old biofilm, which was subsequently incubated for an additional 24 h at 37 °C in 5% CO2, after which the CFU count was determined.

The growth curves were nonlinearly fitted with a 4-parameter equation in GraphPad Prism 9 (GraphPad Software, San Diego, California, USA).

### Confocal laser scanning microscopy (CLSM)

The USA300GFPBas strain was inoculated in medium to an OD_600_ of 0.07, and 500 nM CXCL10, or heat inactivated CXCL10 was added. Biofilm formation was observed for up to 24 h under vital conditions via an inverse confocal laser scanning microscope (LSM980) with excitation at 488 nm (argon the laser line) and a 20 × air objective. The experiments were performed under static conditions in Nunc™ MicroWell™ 96-well plates (Thermo Fischer Scientific) that were placed on a heating device adjusted to 37 °C. An area of 0.829 µm (X) × 0.829 µm (Y) was screened per well at 1 µm Z intervals in the green channel (522 nm). A Z range of 11 µm was defined (11 Z stacks per set), which ensures that the area just above the glass surface where the biofilms forms is captured. The confocal aperture was set to 1 air unit, which corresponds to a confocal aperture of 23 μm. This means that Z stack light is collected over a larger area than just 1 µm, which is also intended to ensure that the biofilms formation level is also recorded in automatic mode. The images were processed using ZEN Blue software (both Carl Zeiss AG, Jena, Thuringia, Germany). The quantity of the biomass was assessed by the ZEN image analysis module, which places the threshold into the automatic modus to reduce noise and maximize the signal output. For the graphical display and statistics, only the results of images with the highest bacterial biomass were used. The GFP signal intensities were plotted against time and fitted with the Gompertz model in GraphPad Prism 9 (GraphPad Software).

### Mass spectrometric analysis

The supernatants of the monocytes challenged with PANG or SpA were supplemented with SDS and dithiothreitol (end concentrations of 1% and 50 mM, respectively), heated for 5 min at 95 °C, and then further diluted in urea buffer (8 M urea, 100 mM Tris-HCl, pH 8.0). Buffer exchange and protein digestion were performed as follows: the reduced proteins were transferred to a 10 kDa Microcon YM-10 filter (Merck KGaA, Darmstadt, Germany) and centrifuged at 14,000 × g for 20 min in all consecutive steps, and the flow-through was discarded. For washing, 200 µL of urea buffer was added, and the centrifugation was repeated. One hundred microliters of alkylation solution (0.1 M iodoacetamide in urea buffer) was added, and the samples were incubated for 20 min in the dark. The alkylation solution was removed by centrifugation followed by two additional centrifugation steps with 200 µL of 8 M urea buffer. Afterward, the samples were washed and centrifuged twice with 200 µL of 50 mM ammonium bicarbonate buffer. Proteins were digested by the addition of 0.5 µg of trypsin in 50 µL of 50 mM ammonium bicarbonate (all Merck KGaA). Proteolytic cleavage was allowed for 16 h at 37 °C, and the peptides were eluted by centrifugation at 14,000 × g for 20 min. To collect residual peptides, the centrifugation was repeated twice after the addition of 50 µL ammonium bicarbonate buffer. The eluted peptides were dried in a SpeedVac (Thermo Fisher Scientific) and reconstituted by adding 25 µL of 0.3% formic acid in water.

Tryptic peptides were analyzed with a Dionex UHPLC coupled to an Orbitrap Fusion LC‒MS/MS system (all Thermo Fisher Scientific). Full mass spectrometry scans were acquired in the Orbitrap (m/z range 370-1,570, quadrupole isolation) at a resolution of 120,000 (full width at half maximum) within 150 min of a nonlinear gradient from 2 to 90% acetonitrile/0.1% formic acid (Merck KGaA). Ions were fragmented by higher-energy collisional dissociation (HCD, 30% collision energy), and a maximum of 20 fragment ion spectra were acquired per cycle in the ion trap in rapid scan mode. The following conditions were used: spray voltage of 2 kV, heated capillary temperature of 275 °C, S-lens RF level of 60%, maximum automatic gain control (AGC) value of 4 × 10^5^ counts for MS1 with a maximum ion injection time of 50 ms and a maximum AGC value of 1 × 10^4^ for MS2, with a maximum ion accumulation time of 35 ms. A dynamic mass exclusion time window of 60 s was set with a 10 ppm maximum mass window. These experiments were independently performed three times.

### Protein identification and quantification

All raw files were searched against the human UniProt database (version 05.2016, reviewed sequences) and the uniparc proteome UP000244076 (*S. aureus* strain ATCC 43300) with MaxQuant version 1.6.17.0 (Max Planck Institute of Biochemistry, Germany). The parameters used were as follows: first search peptide tolerance: 20 ppm; main search peptide tolerance: 4.5 ppm (for MaxQuant); enzyme: trypsin, maximum two missed cleavages; static modification: carbamidomethylation of cysteine residues; variable modifications: methionine oxidation; minimum peptide length: 6, maximum peptide mass: 7600 Da. Normalization was performed in MaxQuant using the label-free quantification (LFQ) setting, with the minimum ratio count set to 2 (unique and razor peptides). Further analysis of LFQ and protein intensities was performed using the Perseus software package version 1.6.2.2 (Max Planck Institute of Biochemistry). The LFQ intensities were log2-transformed (missing values were imputed from the normal distribution of the dataset (width: 0.3, downshift 1.8)). Known contaminants, reverse-identified proteins, and “identified by site” proteins were discarded. Proteins with fewer than two identifications in at least one group were removed from the dataset.

### Flow cytometry analysis

The levels of the chemokine CXCL10 in the supernatants of treated and untreated PBMCs and monocytes were quantified using the bead-based multiplex LEGENDplex™ HU Proinflammatory Chemokine Panel 1 w/VbP (BioLegend, Inc.) according to the manufacturer’s instructions. The samples were measured with an Accuri 6 flow cytometer (BD Biosciences, New Jersey, USA), analyzed with the LegendPlex online server (BioLegend) and compared with standard curves. The experiments were performed in technical duplicates and in six independent biological replicates.

Monocytes incubated with and without PNAG or SpA were stained with monoclonal antibodies conjugated with fluorophores after being boosted for 4 h with phorbol 12-myristate 13-acetate (PMA)/ionomycin (56.08 µg/ml and 1000.88 µg/mL, respectively). The fluorochrome-conjugated monoclonal antibodies (MAbs) PerCP-Cy5.5-anti-CD14 and AF647-anti-CXCL10 (all from BioLegend Inc., San Diego, USA) were used according to the manufacturers’ instructions. The cells were harvested and immediately stained for surface and intracellular antigens. For surface staining, the cells were washed with FACS buffer (HBSS containing 3% FBS and 0.02% sodium azide) (all from Biolegend, Inc.) and incubated with a mixture of 1 µl of each of the monoclonal antibodies at 4 °C in the dark for 30 min. For intracellular staining, the cells were pelleted and resuspended in 150 µL of Cyto-Fast Fix/Perm buffer (BioLegend Inc.), followed by a 20 min incubation at RT. After incubation, the cells were washed with 1 mL of Cyto-Fast perm wash solution and centrifuged at 350 × g for 5 min. The optimal concentrations of intracellular antibodies, prepared in Cyto-Fast perm wash solution, were added to the cells (100 µL total volume) and incubated for 20 min in the dark at 4 °C. After staining, the cells were washed with 1 mL of Cyto-Fast perm wash solution and centrifuged at 350 × g for 5 min. The cells were then washed with 1 mL of FACS wash solution and centrifuged at 350 × g for 5 min. Following the staining and washing steps, the cells were fixed with 1% paraformaldehyde (PFA) (Thermo Fisher Scientific) and analyzed using an LSR Fortessa flow cytometer (BD Biosciences, NJ, USA), with at least 100,000 collected events. The data were analyzed using FlowJo software (Version 10.8.1, Treestar, Ashland, USA).

### Reverse transcription and quantitative PCR

PBMCs challenged with PNAG or SpA were collected in 5-ml round-bottom FACS tubes (BD Vacutainer, USA) and washed with PBS three times. Total RNA was extracted using an RNeasy Mini Kit (Qiagen, Venlo, The Netherlands) following the manufacturer’s instructions. The concentration and purity of the extracted RNA were analyzed using a Nanodrop (A&E Lab, Nano200, UK), and the quality expressed as the RNA integrity number (RIN) was assessed via the Bioanalyzer RNA 6000 Nano assay on an Agilent Bioanalyzer 2100 (all Agilent Technologies, Santa Clara, USA). A RIN > 7 was considered eligible for further processing of the samples.

Primers and probes (Table 1) were designed for the genes encoding the *CXCL10, Dectin-1, CARD9, Syk, PKCδ, NF-kB p65* and *18S rRNA* genes as references. The primers and probes were purchased from Merck KGaA in lyophilized form, resuspended in diethyl dicarbonate (DEPC)-treated water to a final concentration of 10 µM and stored at −20 °C. The primer efficiency was assessed by first performing target-specific PCRs with cDNA, which yielded amplicons corresponding to the genes of interest. qPCR was performed with an overall PCR efficiency of 97.2% (NF-κB: 96.63%; PKCδ: 99.70%; CXCL10: 93.82%; dectin-1: 96.65%; Syk: 99.01%; CARD9: 97.48%. The amplification of amplicons was verified through gel electrophoresis, followed by the excision and purification of specific bands using a gel extraction kit (kit specifications). The quantification of DNA was performed using prior knowledge of the exact fragment sizes of the amplicons. This enabled us to calculate the copy numbers of our target genes on the basis of the measured DNA concentrations. To assess PCR efficiency, we systematically created a dilution series covering a dynamic range from 10^10^ to 10^2^ amplicon copies. This approach was implemented in singleplex reactions, utilizing three replicates for each dilution. Triplicate measurements of the mean threshold cycle (CT) values for each dilution formed the basis for constructing standard curves. These curves, which plot CT values against the logarithm of the target DNA template concentration, served as the basis for PCR efficiency determination. The PCR efficiency (E) was accurately calculated from the slopes (S) derived from the linear regressions of the CT standard curves using the formula E = 10^(-1/slope) – 1^47,48^. A PCR efficiency value of 1 (100%) indicates perfect amplification, where the amount of target DNA doubles in each cycle. An efficiency below 1 suggests suboptimal amplification, which can occur due to factors, such as inhibition, primer–dimer formation, or suboptimal reaction conditions.

**Table 1:** Primers and probes used in this study

Primer	Primer sequence (5‘- 3‘)
CXCL10_For	CTAGAACTGTACGCTGTACC
CXCL10_Rev	TTGATGGCCTTCGATTCTGG
Dectin1_For	ACAATGCTGGCAACTGGGCTCT
Dectin1_Rev	AGAGCCATGGTACCTCAGTCTG
Syk_For	CGTATGAGCCAGAACTTGCACC
Syk_rev	CTTTCGGTCCAGGTAAACCTCC
CARD9_For	TCCGACCTGGAAGATGGCTCAC
CARD9_Rev	CAGAGCTGCAAAGGGCTGTTTC
PKCδ_For	GCTGACACTTGCCGCAGAGAAT
PKCδ_Rev	GCCTTTGTCCTGGATGTGGTAC
NF-kB_For	TGAACCGAAACTCTGGCAGCTG
NF-kB_Rev	CATCAGCTTGCGAAAAGGAGCC
CXCL10_Probe	CTGCAAGCCAATTTTGTCCACG
Dectin1_Probe	CACTTCGACTCTCAAAGCAATACCA
Syk_Probe	CATGGACACAGAGGTGTACGAGA
CARD9_Prob	CCTCACGCATCACACCTTACCT
PKCδ_Probe	GGGACTGGTGAAGCAGGGATT
NF-kB_Probe	CAGGCGAGAGGAGCACAGATAC

cDNA synthesis was performed using 400 ng of total RNA and the Maxima H Minus First Strand cDNA-Synthese Kit (Thermo Fisher Scientific) according to the manufacturer’s instructions, which included random oligohexamers, reverse transriptase and DNAase digestion and inactivation of the enzymes.

For quantitative PCR (qPCR), 2 µl of the reverse transcription mixture and 200 nM of the respective primer pairs per reaction and the iQ™ Multiplex Powermix (Bio-Rad Laboratories, Inc., Feldkirchen, Germany) were used according to the manufacturer’s instructions. In total, 40 cycles were run in the Rotor-Gene Q (Qiagen GmbH), and the fluorophore-tagged probed intensity was determined. The signals that reached the threshold within 36 cycles were considered for quantification. The ΔCt values of each target were assessed in relation to those of 18S rRNA. Therefore, the relative FC in transcripts of the targets was calculated on the basis of the 2^-ΔΔCt^ method in relation to the mean of the untreated controls for the respective experiment.

### Statistics and Reproducibility

The visualization and statistical analysis of the chemokine secretion data obtained from the multiplex cytometric bead arrays and the RT‒qPCR were performed in GraphPad Prism 9. Nonparametric one-way ANOVA with the Kruskal‒Wallis test was used for multiple comparisons of FC and chemokine levels, and *p* values < 0.05 were considered significant. Each experiment was performed with at least three to six biological replicates and two technical replicates. Bacterial growth was analyzed using a non-parametric test for at least three replicates, and growth curves were non-linearly fitted with 4 parameters.

Differences in the expression of the proteins assessed by LC‒MS‒MS/MS were performed in at least 3 biological replicates and analyzed via Perseus application^49^ via a t-test with BH correction. Statistical differences in the protein LFQ abundances were assumed for *q* values < 0.05 (BH-adjusted *p* value). To reduce the data dimension, a principal component analysis was applied to BH-corrected LFQ values using the Perseus software package (see above). To compare the differences in the secretome among the control groups, the -log10_*q*_ values were plotted (volcano plot) against the FC in LFQ abundance as log_FC_. Significantly different secreted proteins were assumed at -log10_*q*_ > 1.3 (corresponding to *q* < 0.05) and log_FC_ > 2 (corresponding to FC < 2). We used the STRING database (Search Tool for the Retrieval of Interacting Genes/Proteins) to visualize the association protein‒protein interaction network and the Markov cluster algorithm clustering option (provided by STRING) to identify functional clusters. The functional relationships of the proteins were assessed based on the GO terms. Here, only the significantly differentially secreted proteins related to biofilms or planktonic stress were utilized. For further details on analysis and experimental design, please refer to the respective sections.

### Reporting summary

Further information on research design is available in the Nature Portfolio Reporting Summary linked to this article.

## Supplementary information

Supplementary material

Reporting summary

## Data Availability

The mass spectrometry proteomics data have been deposited to the ProteomeXchange Consortium via the PRIDE^50^ partner repository with the dataset identifier PXD044120. All individual data supporting the findings of this study are available on the EU open research repository; 10.5281/zenodo.15755652. Custom analysis scripts and any additional information required for data reanalysis or study replication are available from the corresponding author upon reasonable request.
