# Supplementary material for: Staphylococcus aureus biofilm-associated component PNAG stimulates the secretion of the immunomodulatory chemokine CXCL10 via Dectin-1 signaling
Source: Commun Biol. 2025 Jul 31;8:1136. doi: 10.1038/s42003-025-08503-z (PMC12314102; doi:10.1038/s42003-025-08503-z)
Supplement: Supplementary file 1 — Supplementary material [file 42003_2025_8503_MOESM1_ESM.pdf]

Staphylococcus aureus biofilm-associated component PNAG stimulates the secretion of the immunomodulatory chemokine CXCL10 via Dectin-1 signaling

Reza Gheitasi[1]*, Daniel Weiss[1], Mario M. Müller[2,5], Karolin Sommer[1], Daniela Roell[1], Alexander Mosig[3,2], Mathias. W. Pletz[1,4] and Oliwia Makarewicz[1,4]

[1] Institute of Infectious Diseases and Infection Control, Jena University Hospital / Friedrich Schiller University, Jena, 07747, Germany.

[2] Septomics Research Center, Jena University Hospital, Jena, 07747, Germany.

[3] Institute for Biochemistry II, Jena University Hospital / Friedrich Schiller University, Jena, 07743, Germany.

[4] Integrated Research and Treatment Center - Center for Sepsis Control and Care (CSCC), Jena University Hospital, Jena, 07747, Germany.

[5] Functional Proteomics, Jena University Hospital, 07747, Jena, Germany.

Running title: PNAG induces NF-kB and CXCL10

Corresponding author: reza.gheitasi@med.uni-jena.de

Keywords: *Staphylococcus aureus*, biofilm, Poly-*N*-acetylglucosamine, proteomics, NF-kB, chemokine, flowcytometry

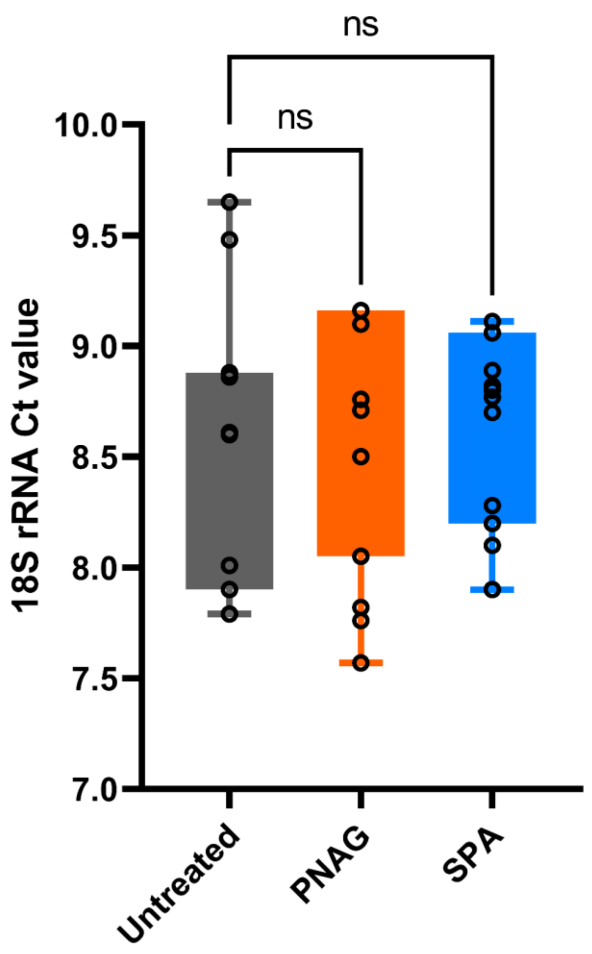

Figure S 1: Ct Values of the 18S rRNA (X-axis) in the PBMCs treated with PNAG or SpA compared to untreated control group. The values are presented as the mean and the standard deviation (SD) of 6 independent experiments. Asterisks indicate significant differences as p values ns ≤ non-significant, and * ≤ 0.05.

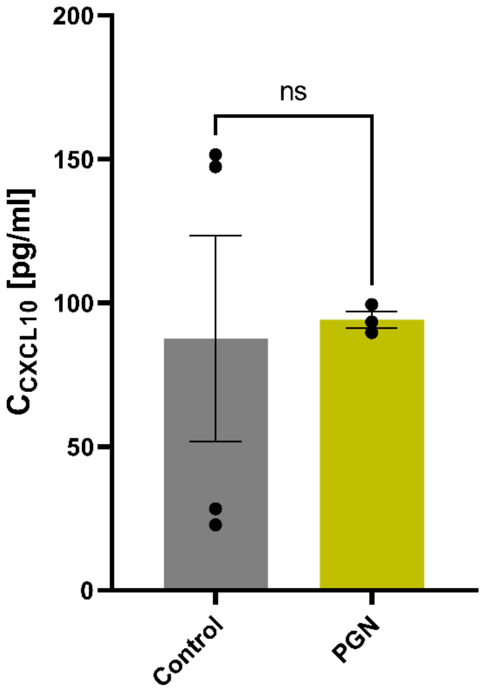

Figure S 2. Effects of PGN on the secretion of CXCL10 (**A**) by PBMCs versus the untreated control. Significance is indicated as follows: ns = non-significant, * $p < 0.05$.

Abbreviations: PGN: S. aureus peptidoglycan

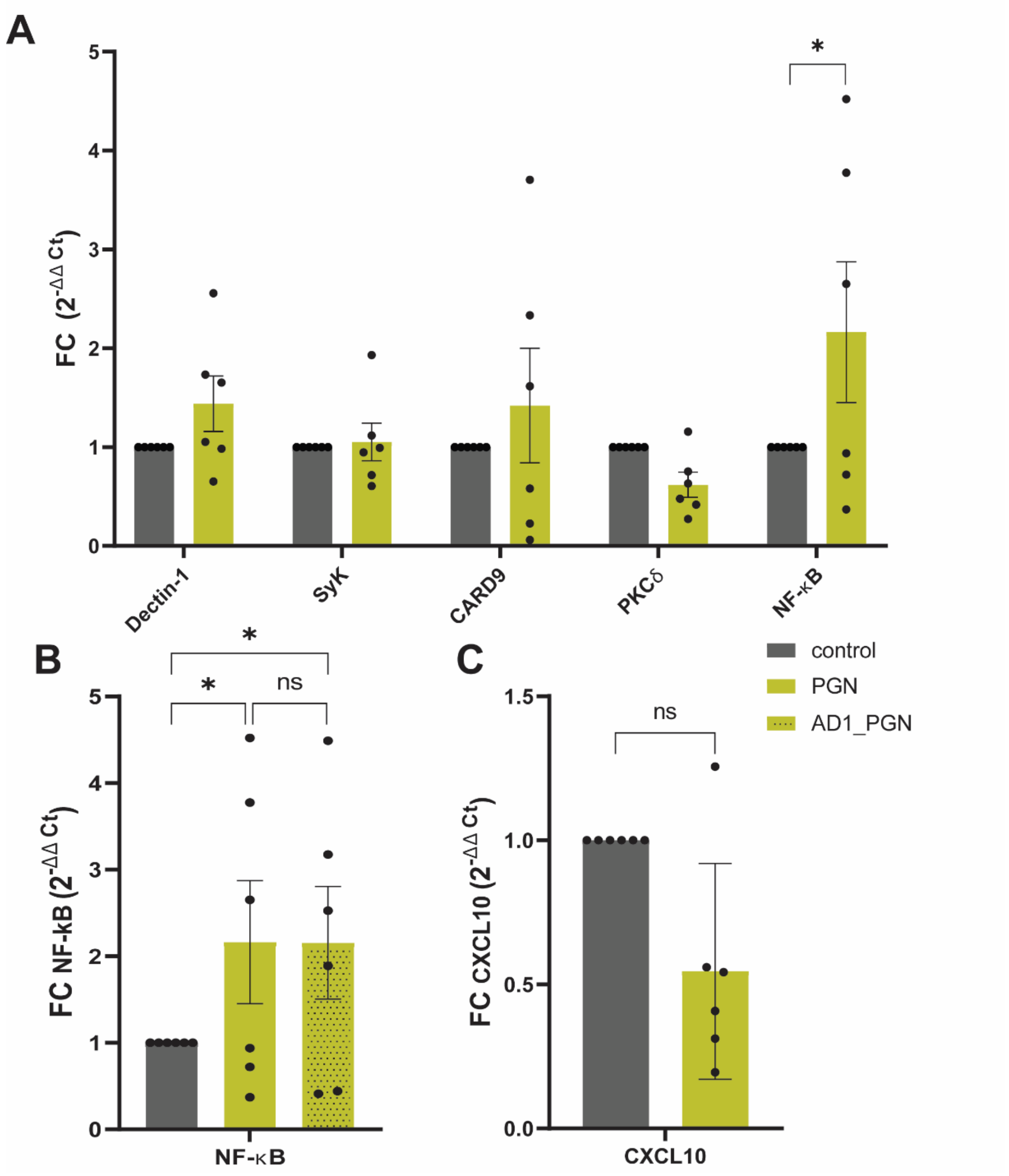

Figure S 3: Relative expression of the selected genes (X-axis) **(A)** in the PBMCs treated with PGN expressed as $2^{-\Delta\Delta Ct}$ normalized to the reference gene. **(B)** Effect of Dectin-1 blockade on NF-κB expression of PBMCs, and **(C)** expression of CXCL10 in PBMCs exposed to PGN compare to untreated PBMCs. The values are presented as the means and standard deviations (SDs) of 3 independent experiments. Significance is indicated as follows: ns = non-significant, * $p < 0.05$. *Abbreviations: PGN: S. aureus peptidoglycan, AD1_PGN: Antibody blocked dectin-1 PBMC treated with peptidoglycan*

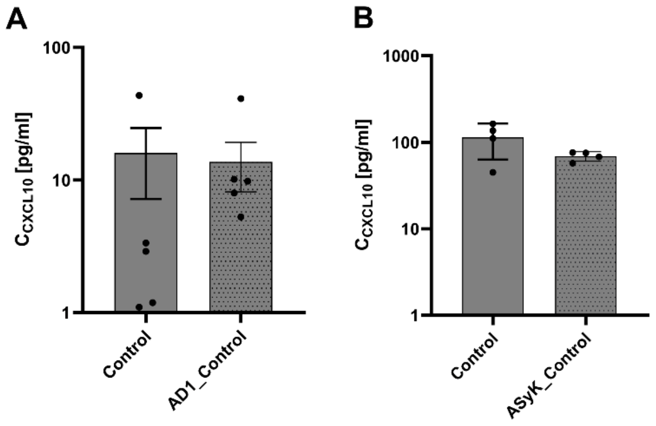

Figure S 4: Effect of Dectin-1 blockade and Syk inhibition on CXCL10 secretion of PBMCs, (**A**) Secretion of CXCL10 with/out antibody blockade against dectin-1 on untreated PBMCs. (**B**) The CXCL10 secreted level of PBMCs with/out inhibition of protein tyrosine kinas SyK. The values are presented as the mean and the standard deviation (SD) of 3 independent experiments. *Abbreviations:* AD1_control: Antibody blocked dectin-1 PBMC, ASyK_control: SyK inhibited PBMC.

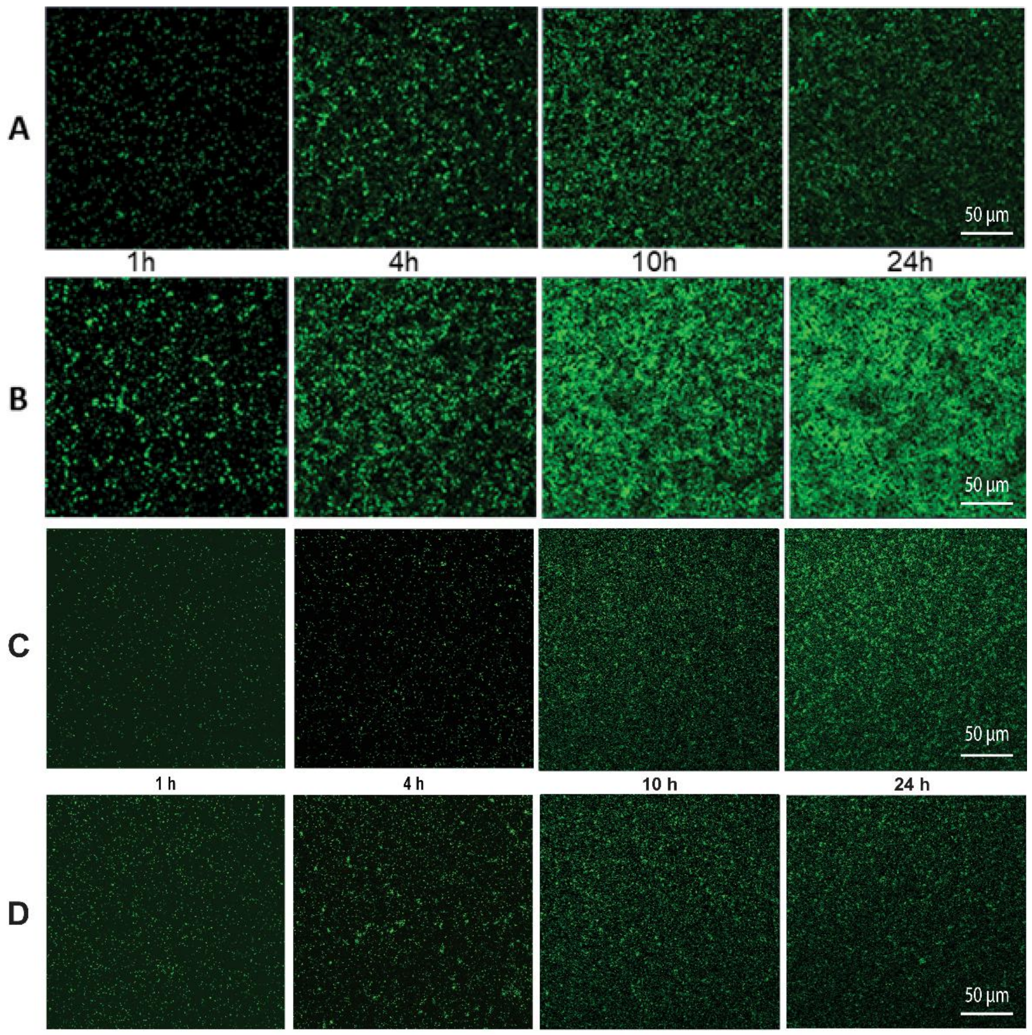

Figure S 5: CLSM visualization of the biofilm formation of GFP-labeled *S. aureus* **(A)** untreated, (**B**) in the presence of 500 nM CXCL10, **(C)** in the presence of 500 nM heat-denatured CXCL10 and (**D**) PBS. Images were recorded via CLSM at 10 × 20 magnification, and the layers with the highest bacterial density within the biofilms were chosen for visualization. Each image shows a 0.829 µm × 0.829 µm section. Of two representative experiments, only the time points 1 h, 4 h, 10 h, and 24 h after the start of biofilm formation are shown.

Table S1: Differentially expressed proteins in a significantly lower or in a higher content when the PBMC were challenged with biofilms compared to the planktonic *S. aureus.*

Gene name	Protein name	*q*-value	Difference
**PBMCs secretome challenged with planktonic *S. aureus***			
HNRNPA2B1	Heterogeneous nuclear ribonucleoproteins A2/B1	0.00122509618384282	-8.10610771179199
HK3	Hexokinase-3	0.00138559797052849	-4.55558904012045
LSM3	U6 snRNA-associated Sm-like protein LSm3	0.00225081976785979	-3.68449211120605
SND1	Staphylococcal nuclease domain-containing protein 1	0.00232003026617817	-3.72782198588054
TPM3		0.00237272466422697	-6.95476849873861
OSTF1	Osteoclast-stimulating factor 1	0.00241571821431637	-4.55930773417155
CUL3	Cullin-3	0.00297950100523013	-2.68819999694824
LMNB2	Lamin-B2	0.00320025507489868	-4.59950574239095
AIF1	Allograft inflammatory factor 1	0.00324897549029276	-3.69183858235677
HNRNPC	Heterogeneous nuclear ribonucleoproteins C1/C2	0.00338688862703733	-5.15377680460612
PDCD6IP	Programmed cell death 6-interacting protein	0.00383067671979529	-4.72972106933594
HCLS1	Hematopoietic lineage cell-specific protein	0.00383170580134592	-4.53725306193034
HSPA4	Heat shock 70 kDa protein 4	0.00397016661393568	-4.87321408589681
NACA	Nascent polypeptide-associated complex subunit alpha, muscle-specific form	0.00397016661393568	-3.76478640238444
MSN	Moesin	0.00397016661393568	-3.49235725402832
HNRNPD	Heterogeneous nuclear ribonucleoprotein D0	0.00402990778790762	-5.79779052734375
GMFG	Glia maturation factor gamma	0.0041987210073388	-4.49427159627279
SH3BP1	SH3 domain-binding protein 1	0.00437467291374116	-3.69127400716146
ECHDC1	Ethylmalonyl-CoA decarboxylase	0.00437467291374116	-2.885955174764
BLVRB	Flavin reductase (NADPH)	0.00474064582308963	-3.57093556722005
SLC9A3R1	Na(+)/H(+) exchange regulatory cofactor NHE-RF1	0.00547121576384012	-4.84702555338542
CRK	Adapter molecule crk	0.00547121576384012	-2.83344841003418
HNRNPA3	Heterogeneous nuclear ribonucleoprotein A3	0.00581641911855921	-2.21422894795736
CAST	Calpastatin	0.00665365209343952	-3.59560648600261
ACTL6A	Actin-like protein 6A	0.00778497518249827	-3.54909451802571
PA2G4	Proliferation-associated protein 2G4	0.00859712725982617	-3.31376393636068
Septin7	Septin-7	0.00894782287908858	-5.48598798116048
HNRNPDL	Heterogeneous nuclear ribonucleoprotein D-like	0.00895410925881499	-3.21225992838542
EEF1D	Elongation factor 1-delta	0.0091010170650809	-4.28445307413737
Tenascin-X	Tenascin-X	0.00929738162703745	-4.20404688517252
RANBP1	Ran-specific GTPase-activating protein	0.00948448416433658	-3.33571434020996

PRKAR1A	cAMP-dependent protein kinase type I-alpha regulatory subunit	0.00977649771193105	-3.46152877807617
NAP1L4	Nucleosome assembly protein 1-like 4	0.0100082590281251	-4.51067733764648
XPO1	Exportin-1	0.0101581959738904	-2.68554433186849
ST13	Putative protein FAM10A4	0.0103952265833467	-4.22672780354818
SRI	Sorcin	0.0104994471138748	-2.5242436726888
SEC23A	Protein transport protein Sec23A	0.01105117914789	-3.47557067871094
NCF1C	Putative neutrophil cytosol factor 1C	0.0123006181719783	-3.57029787699382
HMHA1	Minor histocompatibility protein HA-1	0.0132159145324689	-3.00732040405273
UCHL3	Ubiquitin carboxyl-terminal hydrolase isozyme L3	0.0132948418570147	-3.24047152201334
SRSF1	Serine/arginine-rich splicing factor 1	0.0134321693477346	-2.22571627298991
STK10	Serine/threonine-protein kinase 10	0.0138816852171251	-2.42886861165364
STIP1	Stress-induced-phosphoprotein 1	0.0149877971508931	-2.20783869425456
UBA7	Ubiquitin-like modifier-activating enzyme 7	0.0158781353978281	-3.3315060933431
EIF2S2	Eukaryotic translation initiation factor 2 subunit 2	0.0166947635412399	-3.02618153889974
COPS2	COP9 signalosome complex subunit 2	0.0167952466259627	-1.78138287862142
APOBR	Apolipoprotein B receptor	0.01680710686694	-2.90913263956706
PACSIN2	Protein kinase C and casein kinase substrate in neurons protein 2	0.0170552071562728	-3.32851537068685
EEF1B2	Elongation factor 1-beta	0.0175703507409862	-6.49446296691895
SFPQ	Splicing factor, proline- and glutamine-rich	0.0179598601503734	-3.85586802164714
PSME2	Proteasome activator complex subunit 2	0.0198651481307435	-3.89208348592123
USP14	Ubiquitin carboxyl-terminal hydrolase 14	0.0199065307847321	-3.43786684672038
PCNA	Proliferating cell nuclear antigen	0.0208943512153932	-2.94668579101563
RPRD1B	Regulation of nuclear pre-mRNA domain-containing protein 1B	0.0215541788345109	-2.16319338480632
PABPC1	Polyadenylate-binding protein 1	0.0218196157134224	-3.74851671854655
ADD1	Alpha-adducin	0.0219294089277804	-2.27154223124186
AK2	Adenylate kinase 2, mitochondrial	0.0228134296314419	-4.87518501281738
PDAP1	28 kDa heat- and acid-stable phosphoprotein	0.0228134296314419	-2.93706448872884
USP5	Ubiquitin carboxyl-terminal hydrolase 5	0.0237584607025613	-3.65934308369955
DAK	Bifunctional ATP-dependent dihydroxyacetone kinase/FAD-AMP lyase (cyclizing)	0.0250447963809251	-4.49803733825684
CFL1	Cofilin-1	0.0251997897861103	-3.29045041402181
CNDP2	Cytosolic non-specific dipeptidase	0.0266458795699423	-4.11043039957682
CAPZA1	F-actin-capping protein subunit alpha-1	0.026823108332479	-3.28817176818848
RNH1	Ribonuclease inhibitor	0.0268241437348965	-1.26825777689616
PSMD4	26S proteasome non-ATPase regulatory subunit 4	0.0271481128981753	-1.82021649678548
WAS	Wiskott-Aldrich syndrome protein	0.0278833141371528	-3.33872667948405
PCBP1	Poly(rC)-binding protein 1	0.0297264843622919	-3.31076367696127
MTPN	Myotrophin	0.0299691658799691	-5.6730162302653
ENO1	Alpha-enolase	0.0320825341237851	-1.18041928609212
LZIC	Protein LZIC	0.0324192030522067	-4.23792394002279
UFM1	Ubiquitin-fold modifier 1	0.0324192030522067	-3.21223704020182
TXNL1	Thioredoxin-like protein 1	0.0329158070126917	-3.00758870442709

PRDX5	Peroxiredoxin-5	0.0329335109103681	-5.48060862223307
APBB1IP	Amyloid beta A4 precursor protein-binding family B member 1-interacting protein	0.0329335109103681	-1.75470288594564
HNRNPK	Heterogeneous nuclear ribonucleoprotein K	0.0331336808820948	-2.84608713785807
IL16	Interleukin-16	0.0351401202336949	-3.82675806681315
PGK1	Phosphoglycerate kinase 1	0.0364064560643602	-3.61030197143555
RBM8A	RNA-binding protein 8A	0.0364064560643602	-3.31323051452637
IST1	IST1 homolog	0.0365839080826411	-2.97500356038411
ZYX	Zyxin	0.0384886044804208	-5.33604685465495
SAFB2	Scaffold attachment factor B2	0.03870437507837	-2.86786460876465
HSPB1	Heat shock protein beta-1	0.0394799109341325	-4.79301261901855
PPA1	Inorganic pyrophosphatase	0.0417428300126576	-2.61240450541178
CCT8	T-complex protein 1 subunit theta	0.0422651203330631	-2.83065223693848
**PBMCs secretome challenged with *S. aureus* biofilm**			
NFS1	Cysteine desulfurase	0.00122509618384282	4.57199096679688
DPP7	Dipeptidyl peptidase 2	0.00122509618384282	3.44661649068196
PRPF8	Pre-mRNA-processing-splicing factor 8	0.00122509618384282	2.38769467671712
NDUFV2	NADH dehydrogenase [ubiquinone] flavoprotein 2	0.00122509618384282	1.47683525085449
GIMAP7	GTPase IMAP family member 7	0.00125629237862989	1.59690539042155
TES	Testin	0.00173345808679002	3.74503135681152
GGCT	Gamma-glutamylcyclotransferase	0.00179258164364405	4.65143330891927
RWDD1	RWD domain-containing protein 1	0.00222940224916641	2.76996676127116
HSPA9	Stress-70 protein, mitochondrial	0.00268723171806932	8.80506833394368
RPS8	40S ribosomal protein S8	0.00268723171806932	2.73138745625814
ACTG1		0.00270854196253852	2.88363965352377
MAN2B1	Lysosomal alpha-mannosidase;Lysosomal alpha-mannosidase A,B,C,D,E peptides	0.00355508734640218	3.00924301147461
DLST	Dihydrolipoyllysine-residue succinyltransferase component of 2-oxoglutarate dehydrogenase complex	0.00402990778790762	3.78886985778809
DLAT	Acetyltransferase component of pyruvate dehydrogenase complex	0.00402990778790762	3.04300753275553
NIPSNAP3A	Protein NipSnap homolog 3A	0.00419524192517497	4.33978716532389
EFHD2	EF-hand domain-containing protein D2	0.00706683290479033	0.947721481323242
RPS2	40S ribosomal protein S2	0.00936505003372187	2.43293698628743
TRPV3	Transient receptor potential cation channel subfamily V member 3	0.00939019774921722	4.19886589050293
HSP90AB1	Heat shock protein HSP 90-beta	0.00941346035489018	1.10247993469238
FAHD1	Acylpyruvase FAHD1	0.0125047032092976	2.57362492879232
TSNAX	Translin-associated protein X	0.0135848123766292	1.96796417236328
AP3B1	AP-3 complex subunit beta-1	0.0139331305578253	2.88084093729655
HIST2H3PS2	Histone H3	0.0154727508092478	4.84566307067871
MDH2	Malate dehydrogenase, mitochondrial	0.0165837151503223	1.65378252665202
HSP90AA1	Heat shock protein HSP 90-alpha	0.0175320984445254	1.18663597106934
EIF5B	Eukaryotic translation initiation factor 5B	0.0182399369205337	1.70312436421712
RPL22	60S ribosomal protein L22	0.0190231460563828	2.80422655741374
PRKDC	DNA-dependent protein kinase catalytic subunit	0.0237584607025613	3.03001085917155

RPS3	40S ribosomal protein S3	0.025850393318349	3.23059844970703
Septin11	Septin-11	0.0268241437348965	2.41893132527669
U2AF2	Splicing factor U2AF 65 kDa subunit	0.0271481128981753	1.97892634073893
DPYSL2	Dihydropyrimidinase-related protein 2	0.0351401202336949	0.886943817138672
SERPINA3	Alpha-1-antichymotrypsin	0.0365839080826411	1.44390869140625

Table S2: Differentially expressed proteins in a significantly lower or in a higher content when the PBMC were challenged with PNAG compared to the SpA.

Protein name	Gene name	p-value	Difference
**Monocytes secretome challenged with PNAG**			
MIP-1-beta	CCL4	0,00308964321718299	7,99176915486654
Tumor necrosis factor	TNF	0,00522588983943391	4,8984800974528
MIP-1-alpha	CCL3	0,00522588983943391	8,18212890625
ATPase ASNA1	ASNA1	0,00522588983943391	4,3088862101237
Interleukin-8	CXCL8	0,00961041994361642	3,80459912618002
Interleukin-1 beta	IL1B	0,0145445149494108	7,93753496805827
Interleukin-6	IL6	0,0210177261091507	7,28898048400879
Histone H3.1	HIST1H3A	0,0210177261091507	7,65345509847005
Probable ATP-dependent RNA helicase DDX17	DDX17	0,0232031986079654	3,8545118967692
UPF0687 protein C20orf27	C20orf27	0,0232031986079654	5,2452138264974
Target of Myb protein 1	TOM1	0,028560865559665	2,21022415161133
Ferritin heavy chain	FTH1	0,028560865559665	5,53818957010905
Pentraxin-related protein PTX3	PTX3	0,0343086882096186	6,71621831258138
C-C motif chemokine 3-like 1	CCL3L1	0,0359095327384376	6,88743336995443
Fructose-bisphosphate aldolase B	ALDOB	0,0387759100913991	4,14786148071289
Caspase-1	CASP1	0,0459480932353934	4,2295716603597
**Monocytes secretome challenged with SpA**			
Carboxypeptidase	CTSA	0,0136065024782781	-3,62541198730469
Hemoglobin subunit beta	HBB	0,0210177261091507	-9,65143458048502
Hemoglobin subunit alpha	HBA1	0,028560865559665	-6,92162195841471
Transient receptor potential cation channel subfamily V member 3	TRPV3	0,0343086882096186	-4,15073903401693
Neurogenic locus notch homolog protein 3	NOTCH3	0,0343086882096186	-3,33526357014974
Calreticulin	CALR	0,0343086882096186	-4,69311714172363
